# Altered chaperone–nonmuscle myosin II interactions drive pathogenicity of the *UNC45A* c.710T>C variant in osteo-oto-hepato-enteric syndrome

**DOI:** 10.1172/jci.insight.185508

**Published:** 2025-03-24

**Authors:** Stephanie Waich, Karin Kreidl, Julia Vodopiutz, Arzu Meltem Demir, Adam R. Pollio, Vojtěch Dostál, Kristian Pfaller, Marianna Parlato, Nadine Cerf-Bensussan, Rüdiger Adam, Georg F. Vogel, Holm H. Uhlig, Frank M. Ruemmele, Thomas Müller, Michael W. Hess, Andreas R. Janecke, Lukas A. Huber, Taras Valovka

**Affiliations:** 1 Institute of Cell Biology, Biocenter, and; 2 Department of Paediatrics I, Medical University of Innsbruck, Innsbruck, Austria.; 3 Division of Paediatric Pulmonology, Allergology and Endocrinology, Department of Paediatrics and Adolescent Medicine, Comprehensive Center for Paediatrics, Medical University of Vienna, Vienna, Austria.; 4 Vienna Bone & Growth Center (VBGC), Medical University of Vienna, and full member of European Reference Network on Rare Bone Diseases, Vienna, Austria.; 5 Ankara Child Health and Diseases, Training and Research Hospital, Department of Paediatric Gastroenterology, Ankara, Turkey.; 6 Division of Paediatric Gastroenterology, Hepatology and Nutrition, Department of Paediatrics, Ankara University School of Medicine, Ankara, Turkey.; 7 Institute of Histology and Embryology, Medical University of Innsbruck, Innsbruck, Austria.; 8 Université Paris Cité, Laboratory of Intestinal Immunity, Institut IMAGINE INSERM UMR 1163, Paris, France.; 9 University Children’s Hospital, Paediatric Gastroenterology, Hepatology and Nutrition, Medical Faculty Mannheim, Heidelberg University, Mannheim, Germany.; 10 Experimental Medicine Division, Nuffield Department of Clinical Medicine; Department of Paediatrics; and Oxford Biomedical Research Centre, University of Oxford, Oxford, United Kingdom.; 11 Université Paris Cité, Faculté de Santé, UFR de Médicine, APHP, Hôpital Universitaire Necker Enfants Malades, Service de Gastroentérologie Pediatrique, Institut IMAGINE INSERM UMR 1163, Paris, France.; 12 Institute of Human Genetics, Medical University of Innsbruck, Innsbruck, Austria.

**Keywords:** Cell biology, Genetics, Chaperones, Genetic diseases

## Abstract

The osteo-oto-hepato-enteric (O2HE) syndrome is a severe autosomal recessive disease ascribed to loss-of-function mutations in the Unc-45 myosin chaperone A (*UNC45A*) gene. The clinical spectrum includes bone fragility, hearing loss, cholestasis, and life-threatening diarrhea associated with microvillus inclusion disease–like enteropathy. Here, we present molecular and functional analysis of the *UNC45A* c.710T>C (p.Leu237Pro) missense variant, which revealed a unique pathogenicity compared with other genetic variants causing UNC45A deficiency. The UNC45A p.Leu237Pro mutant retained chaperone activity, prevented myosin aggregation, and supported proper nonmuscle myosin II (NMII) filament formation in patient fibroblasts and human osteosarcoma (U2OS) cells. However, the mutant formed atypically stable oligomers and prevented chaperone-myosin complex dissociation, thereby inhibiting NMII functions. Similar to biallelic UNC45A deficiency, this resulted in impaired intracellular trafficking, defective recycling, and abnormal retention of transferrin at various endocytic sites. In particular, coexpression of wild-type protein attenuated the pathogenic effects of the variant by inhibiting excessive oligomer formation. Our results elucidate the pathogenic mechanisms and recessive characteristics of this variant and may aid in the development of targeted therapies.

## Abstract 2

The manuscript provides a thorough molecular and functional analysis of the pathogenic non-muscle myosin co-chaperone *UNC45A* c.710T>C missense variant associated with early-onset Osteo-Oto-Hepato-Enteric (O2HE) Syndrome.

## Introduction

The osteo-oto-hepato-enteric (O2HE) syndrome (OMIM #619377), which combines congenital secretory diarrhea, cholestasis, hearing impairment, and bone fragility, has recently been linked to loss-of-function mutations in the Unc-45 myosin chaperone A (*UNC45A*) gene, encoding a myosin cochaperone (1). Mild intellectual disability and developmental delay occurred in some of the patients with O2HE syndrome. The complex molecular mechanisms underlying this severe neonatal disease are the subject of intense research, and it is still incompletely understood how specific *UNC45A* mutations cause the disease (2–4). Recently, we have shown that loss of UNC45A chaperone activity on the myosin motor protein VB (MYO5B) causes abnormal apicobasal vesicle trafficking in enterocytes and promotes enteropathies in the O2HE syndrome (5). However, the heterogeneity of clinical symptoms and the diversity of histological and subcellular features in the 12 patients with O2HE syndrome reported to date suggest the involvement of multiple UNC45A substrates and dependent processes.

As a member of the highly conserved UCS (UNC45, CRO1, She4p) domain chaperone family, human UNC45A (OMIM *611219) is essential for nonmuscle myosin maturation, assembly, and function (6–10). The human *UNC45A* gene encodes a 944–amino acid myosin cochaperone (NM_018671.5 → NP_061141.2 protein unc-45 homolog A isoform 2) with 4 functionally distinct elements: an N-terminal tetratricopeptide repeat (TPR) domain (1–143 aa), a central domain (144–385 aa), a neck domain (386–557 aa), and a C-terminal UCS domain (558–944 aa) (5), all composed of a series of α-helices connected by loops. At the NH2-terminus, 3 TPR motifs provide binding sites for the heat shock proteins 70 and 90 (Hsp70 and Hsp90) partner chaperones (11). The TPR domain is followed by a rigid armadillo (ARM) repeat-containing central domain. Similar to the homologous UNC45B chaperone, the central domain of UNC45A is thought to stabilize the protein structure by constraining the relative orientation of the most flexible TPR and UCS domains (8). The central domain also promotes the formation of myosin-chaperone complexes and the release of the chaperone from the folded substrates by interacting with distinct sites on the myosin head (12, 13). Folding itself is mediated by the COOH-terminal UCS domain, which binds with high affinity to the unfolded myosin head and is sufficient to prevent its misfolding and thermal aggregation (12–14). UCS and central domains are linked by a neck region. This region tethers TPR domain helices of adjacent molecules, resulting in the assembly of short, polar UNC45A chains of transient nature. Together with Hsp70 and Hsp90, these UNC45A oligomers provide a scaffold that is optimal for efficient binding, spatial placement, nascent myosin folding, and precise filament organization (8, 14).

In nonmuscle cells, bipolar assemblies of actin and nonmuscle myosin II (NMII) form multifunctional structures called stress fibers (15–17). These contractile actomyosin bundles require coordinated NMII folding and filament assembly, which are largely facilitated by UNC45A. Absence of the myosin cochaperone leads to misfolding and aggregation of NMII and to abnormal shape and impaired maturation of focal adhesions of UNC45A-deficient human osteosarcoma (U2OS) cells (18). In addition to facilitating various NMII-associated processes, UNC45A promotes the folding of myosin IB (MYO1B) and MYO5B, supporting their functions in vesicle trafficking (2, 5, 19, 20). Consistent with its role as a dynamic chaperone component of the actomyosin network and a regulator of myosin-dependent cargo transport, *UNC45A* was identified as a gene involved in transferrin (Tfn) and epidermal growth factor endocytosis (21).

We have previously shown that *UNC45A* mutations are responsible for microvillus inclusion disease–like (MVID-like) enteropathy with clinical symptoms of variable severity. Among the published patients with O2HE and biallelic *UNC45A* mutations, 4 patients of Turkish and Chinese ancestry carried the c.710T>C variant on 1 or both alleles (5, 22). The mutation leads to the p.Leu237Pro amino acid substitution within the central domain of the UNC45A chaperone (5). In contrast with other cases, we have found that this mutation does not lead to the loss of the protein. However, the severe course of disease in affected individuals suggests high pathogenicity, and a recent study in another affected infant confirms the increased frequency of this specific *UNC45A* variant (22).

We show here that the UNC45A p.Leu237Pro variant disrupts the normal chaperone-myosin interaction, maintaining chaperone activity but abnormally increasing complex formation with NMII. This leads to impaired intracellular trafficking, mirroring the phenotype observed with UNC45A deficiency. The pathogenic effects of the mutation result from altered chaperone interactions rather than a complete loss of chaperone function, highlighting its recessive nature. This understanding could guide the development of targeted therapies for affected individuals, particularly through the creation of small molecule drugs or peptides. Such therapeutic agents could aim to restore or modify the interactions between the mutant protein and its myosin substrates, potentially correcting the dysfunctional protein behavior and alleviating disease symptoms.

## Results

### The UNC45A c.710T>C missense variant causes O2HE syndrome in independent families.

**Figure 1:**
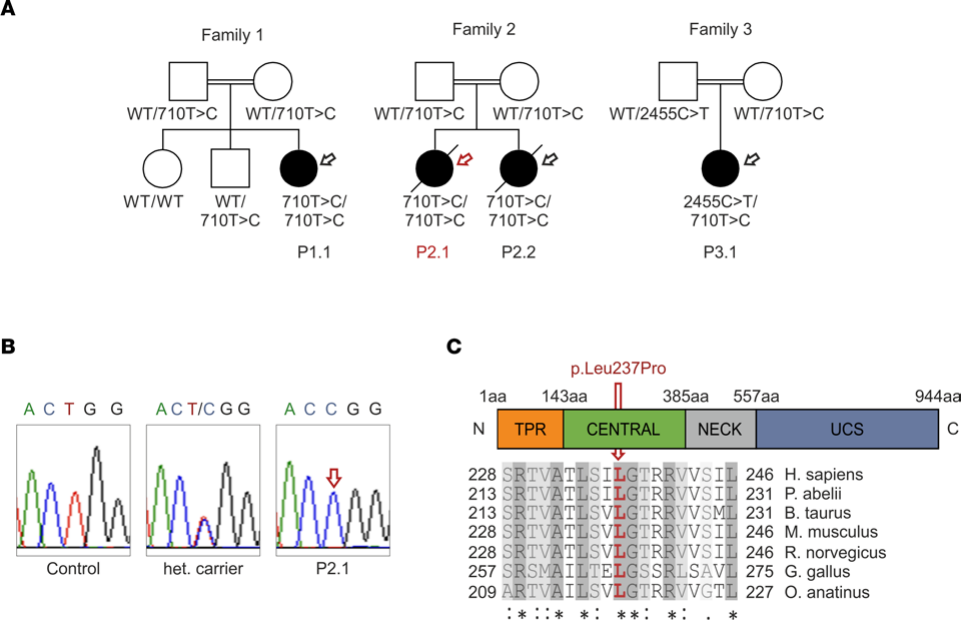
The O2HE syndrome–associated *UNC45A* c.710T>C missense variant is identified in 4 infants from unrelated families. (**A**) The pedigrees of the families studied show the segregation of the *UNC45A* c.710T>C missense variant. Affected individuals are represented by filled symbols, while unaffected individuals are represented by unfilled symbols. Circles and squares denote females and males, respectively. Family 1 and family 2 were part of the patient cohort described in Duclaux-Loras et al., 2022 (5). The red arrow indicates index patient P2.1 in this study. Family 3 was described by Kong et al., 2023 (22), with a patient showing a compound heterozygous genotype. (**B**) Sanger sequencing chromatograms depicting the *UNC45A* c.710T site in a healthy, unrelated individual (control), a heterozygous individual from family 2, and patient P2.1. (**C**) Schematic representation of the UNC45A protein (Q9H301) domain structure. A sequence alignment of UNC45A orthologs from different species shows the high evolutionary conservation of leucine 237 (red arrow). Identical residues are depicted by asterisks (*) and conservative and semiconservative amino acid substitutions by colons (:) and dots (.), respectively.

Next-generation sequencing identified the c.710T>C variant in the *UNC45A* gene (National Center for Biotechnology Information [NCBI] RefSeqGene NG_061633.1) in patients from unrelated families of Turkish and Chinese ancestry. These 4 patients displayed the variant either in homozygous state or compound heterozygous state, with the *c.2455C>T* (p.Arg819Ter) null mutation (5, 22) (Figure 1A). One patient (P1.1) is still dependent on parenteral nutrition at age 5 years, and a pair of siblings (P2.1 and P2.2) presented with O2HE syndrome, with cholestasis, bone fragility, and intractable diarrhea with histopathological features suggestive of MVID (19), and succumbed to multiple organ failure in the first few months of life, indicating severe disease resulting from this mutation (Table 1). The *UNC45A* c.710T>C variant segregated with disease in all examined families (Figure 1, A and B).

The *UNC45A* c.710T>C variant results in the substitution of a highly conserved leucine residue with a proline (p.Leu237Pro) within the central domain of the chaperone (Figure 1C). This variant was considered pathogenic based on the high conservation of the mutated residue, the overlap of clinical symptoms in unrelated individuals, its low frequency of 0.0005% in the Genome Aggregation Database population database, and the familial segregation.

### The UNC45A c.710T>C missense variant does not affect the stability and expression of the UNC45A protein.

**Figure 2:**
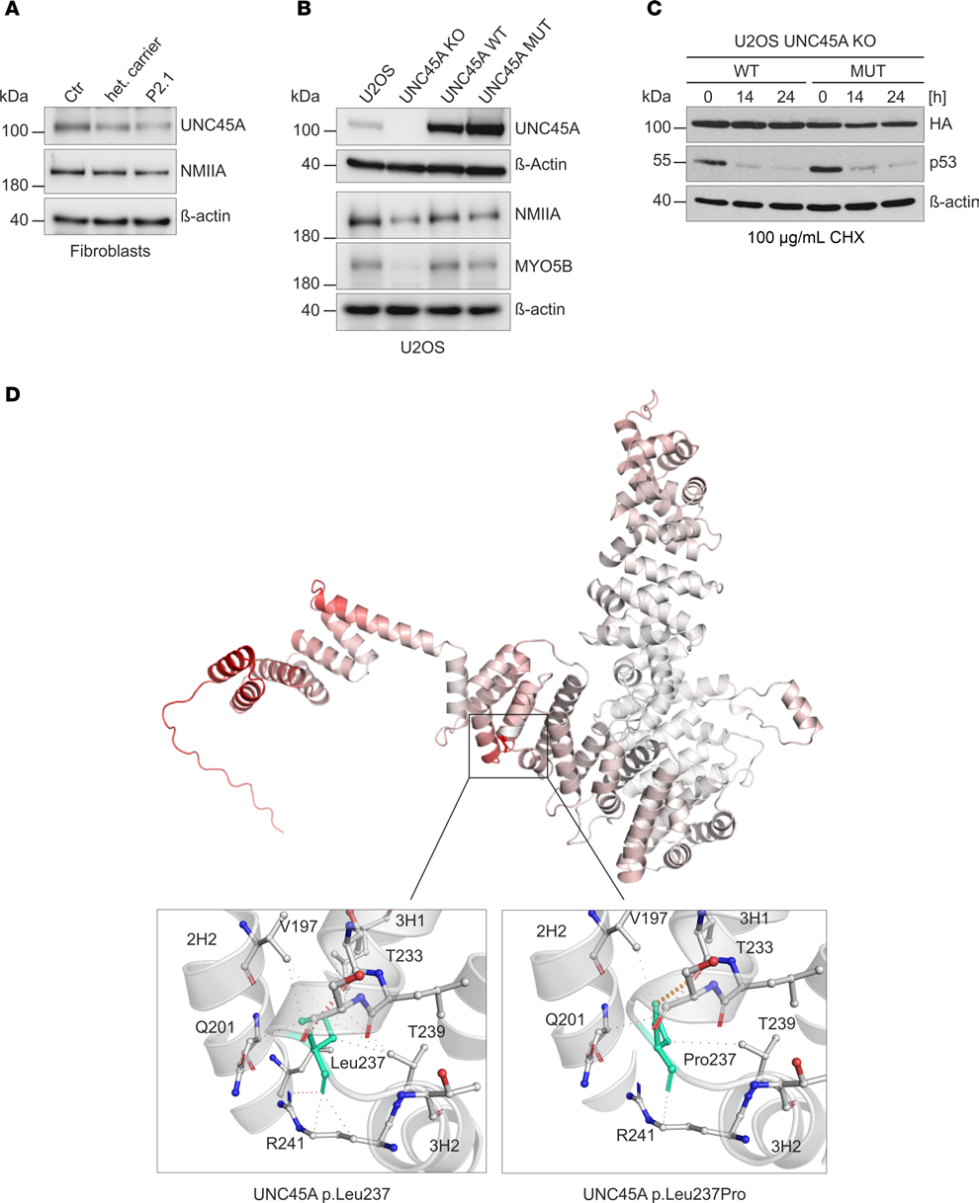
Analysis of UNC45A p.Leu237Pro variant expression, stability, and in silico prediction of structural changes. (**A**) Immunoblot analysis of endogenous UNC45A and NMIIA in fibroblasts originating from skin biopsies of P2.1, a healthy heterozygous family member, and a nonrelated individual (Ctr). β-Actin served as a loading control. (**B**) Parental U2OS, UNC45A-KO, and stably complemented UNC45A wild-type and mutant cells were analyzed for endogenous and ectopic UNC45A expression by immunoblotting. The protein levels of selected myosins were tested using NMIIA- and MYO5B-specific antibodies, and β-actin was used to control the loading. (**C**) U2OS UNC45A-KO cells were transiently transfected with HA-tagged UNC45A wild-type and mutant constructs and treated with 100 μg/mL cycloheximide (CHX) for the indicated times. UNC45A levels were analyzed using HA-specific antibody with p53 levels as a control for CHX treatment and β-actin as a protein loading control. (**D**) Ribbon representation of the human UNC45A protein structure predicted by AlphaFold (accession code: AF-Q9H3U1-F1; upper panel). Vibrational entropy changes (ΔΔSVib) between wild-type and mutant are indicated in red and represent a gain in structural flexibility. Differences in noncovalent intramolecular interactions in wild-type and mutant UNC45A are visualized in the lower panels. Both Leu237 and Pro237 residues are colored in green. The colors of the contacts are based on the following: red and orange, hydrogen and weak hydrogen bonds, respectively; yellow, mixed ionic van der Waals interactions; green, hydrophobic contacts. The residues involved in intramolecular interactions and corresponding α-helices are numbered.

While truncating mutations in *UNC45A* lead to loss of UNC45A protein because of transcript decay, 2 previously reported missense mutations resulted in a substantial reduction in protein levels (1). In contrast, the c.710T>C (p.Leu237Pro) variant in T lymphoblastic cells from patient P1.1 showed protein levels comparable to those of control cells. Furthermore, when this variant was ectopically expressed in HEK293T cells, protein levels remained similar to control (5). We observed comparable expression of UNC45A protein in fibroblasts isolated from skin biopsies of patient P2.1 homozygous for c.710T>C, a healthy heterozygous family member, and healthy controls. Notably, we also observed unchanged expression of the UNC45A substrate NMIIA in patient fibroblasts (Figure 2A).

We corroborated these results in a U2OS UNC45A-knockout (UNC45A-KO) cell line that has been previously used as a model for UNC45A chaperone functions (18). These cells display consistently lower NMIIA and MYO5B levels compared with parental cells because of misfolding and increased degradation (Figure 2B). When these cells were genetically reconstituted with either wild-type UNC45A or the missense variant c.710T>C (p.Leu237Pro), similar levels of ectopic UNC45A protein were detected. These reconstituted cells will be referred to as UNC45A WT and UNC45A MUT, respectively. In addition, both UNC45A constructs were able to effectively rescue NMIIA and MYO5B levels in these cells, verifying our results in patient fibroblasts. To exclude the direct effect of p.Leu237Pro mutation on UNC45A protein stability, we performed a cycloheximide (CHX) chase assay in transiently transfected U2OS UNC45A-KO cells. Treatment with 100 μg/mL CHX drastically reduced p53 protein levels, while wild-type and mutated UNC45A protein levels were not affected (Figure 2C). We conclude that the *UNC45A* c.710T>C missense variant has a negligible effect on the stability and expression of the UNC45A p.Leu237Pro protein and its NMII substrate.

Analysis of the 3D structural model of human UNC45A from the AlphaFold protein structure database (AFBD: AF-Q9H3U1-F1) revealed that Leu237 constituted the C-terminus of helix 1 of the third ARM repeat (3H1) located in the central domain. Leu237 was mutagenized to proline using the DynaMut server to predict the structural effects of the mutation (23). We found that the p.Leu237Pro amino acid exchange had only a minor destabilizing effect leading to a free energy change of –0.152 kcal/mol, as predicted by the Elastic Network Contact Model (ENCoM) method (24). The p.Leu237Pro variant was also found to increase overall structural flexibility according to computed changes in vibrational entropy energy between the wild-type and mutant proteins (ΔΔSVib = 0.190 kcal/mol/K) (Figure 2D). The strongest effect occurred in the central domain, especially in the regions close to the p.Leu237Pro mutation (helices 2H2 and 3H1), as well as in the TPR region and, to a lesser extent, in the C-terminus of the UCS domain (Figure 2D). To explain these effects, we have analyzed the changes in the interatomic interactions imposed by the p.Leu237Pro mutation. The side chain of Leu237 is predicted to form hydrophobic interactions with several residues located in 3H1 and adjacent helices 2H2 and 3H2. In addition, its carbonyl group tends to build a hydrogen bond with the guanidine group of Arg241 in helix 3H2, and the amide nitrogen forms a strong hydrogen bond with the carbonyl oxygen of Thr233. Mutation to proline resulted in the loss of several stabilizing hydrophobic interactions and was incompatible with Arg241- and Thr233-mediated hydrogen bonding (Figure 2D). These results indicate that the Leu237Pro substitution is likely to induce structural changes in different regions of the UNC45A protein. While these changes do not affect the expression and overall stability of the protein, they may affect other functional properties of the mutant UNC45A chaperone.

In addition to the in silico analysis, we assessed heat-induced aggregation of wild-type and Leu237Pro UNC45A proteins ectopically expressed in U2OS UNC45A-KO cells using the cell lysate thermal shift assay, a modified version of the CETSA (25, 26). The assay is based on the concept that a protein unfolds and aggregates when heated to a specific temperature, known as aggregation temperature (T_agg_) (27). Thermal unfolding of proteins can be altered by amino acid substitutions and changes in interatomic interactions, causing a shift in T_agg_, also referred to as thermal shift (28, 29). The thermal aggregation profile of HA-tagged UNC45A WT was similar to that of endogenous protein (30), with the T_agg_ calculated at 50.9°C (Supplemental Figure 1; supplemental material available online with this article; https://doi.org/10.1172/jci.insight.185508DS1). The Leu237Pro variant considerably lowered the thermal resistance of UNC45A, resulting in a T_agg_ of 48.3°C. These results support the predicted loss of stabilizing interactions due to the Leu237Pro substitution in the central domain of the UNC45A chaperone and highlight the pronounced impact of this mutation on protein structure.

### UNC45A p.Leu237Pro acts as an active chaperone and supports NMII folding and actomyosin filament assembly.

**Figure 3:**
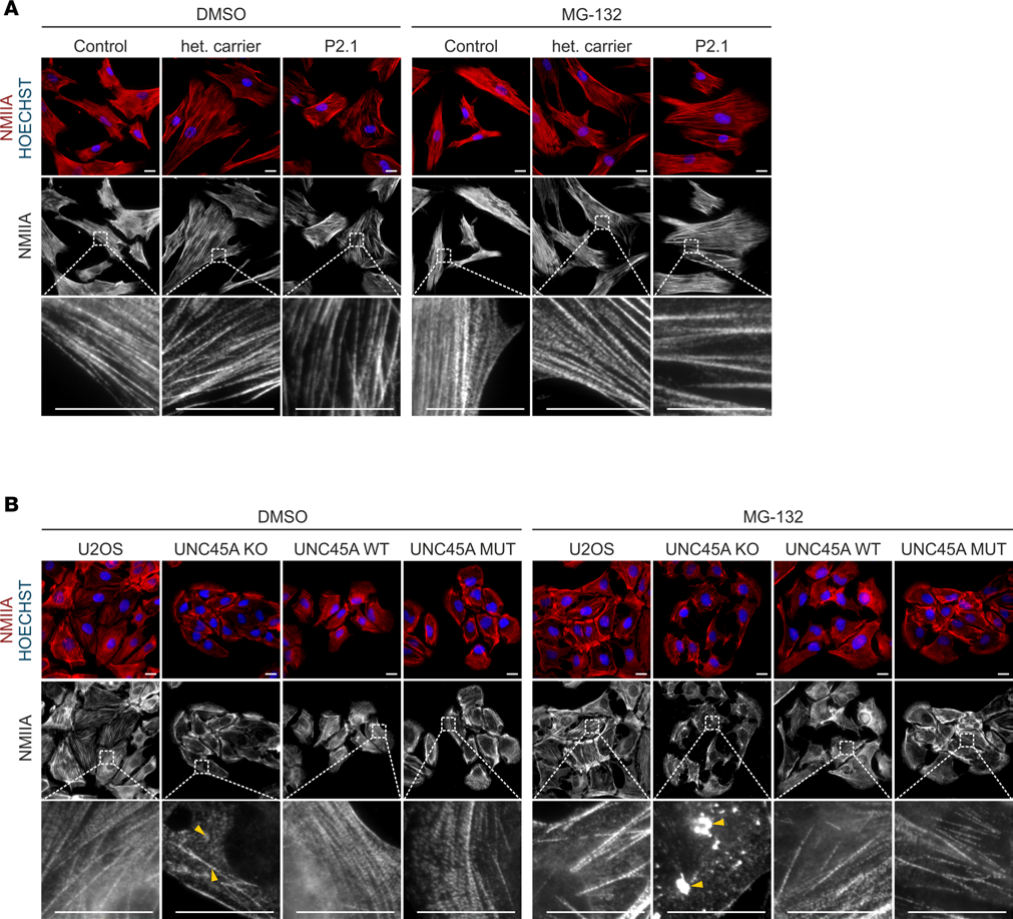
Epifluorescence microscopy showing the UNC45A mutant to retain chaperone activity and support NMIIA folding and assembly. (**A**) Patient P2.1 and control fibroblasts were treated either with vehicle (DMSO) or with 10 μM MG-132 proteasome inhibitor for 4 hours and stained with an antibody recognizing NMIIA. Normal NMIIA patterns were seen under both experimental conditions in all genotypes. Representative image sections were 10× magnified. Nuclei were detected with HOECHST. Scale bars, 20 μm. (**B**) U2OS parental, UNC45A-KO, and stably complemented UNC45A wild-type and mutant cells, treated with 10 μM MG-132 for 4 hours or left untreated, were labeled with an NMIIA-specific antibody (red) and a nucleus marker (HOECHST; blue). Representative image sections were 10× magnified. Yellow arrowheads in untreated UNC45A-KO cells indicate faint and dispersed NMIIA staining and fragmented NMIIA-positive bundles. MG-132–treated UNC45A-KO cells exhibit NMIIA-specific aggregates (yellow arrowheads). In p.Leu237Pro UNC45A–expressing cells, NMIIA filament assembly was rescued to a large extent, and no NMIIA-positive aggregates appeared upon proteasome inhibition. Scale bars, 20 μm.

UNC45A folds and stabilizes NMII through its chaperone activity and assists in the assembly of actomyosin complexes. Both of these functions are mediated by distinct UNC45A domains and are essential for the formation and arrangement of fully functional stress fibers (18). This prompted us to investigate whether the *UNC45A* c.710T>C variant affects NMII folding and filament assembly activities. Immunofluorescence (IF) microscopy with NMIIA-specific antibodies showed regular NMII-associated stress fibers in the patient’s fibroblasts, comparable to those observed in the unrelated donor and the healthy heterozygous carrier (Figure 3A). We investigated the chaperone function of the p.Leu237Pro mutant by treating patient and control fibroblasts with the 26S proteasome inhibitor MG-132. Loss of UNC45A leads to degradation of nascent and misfolded NMII by the 26S proteasome, and blocking this degradation pathway can lead to myosin aggregation (18). We showed that treating patient and control fibroblasts with 10 μM MG-132 did not induce NMIIA aggregates, and the NMIIA network remained virtually unaltered in these cells (Figure 3A). Together, we concluded that NMII folding and filament assembly activities are retained in the p.Leu237Pro UNC45A variant.

To further substantiate these findings, we analyzed the NMIIA network organization in parental, UNC45A-KO, and ectopically complemented U2OS cell lines using IF microscopy (Figure 3B and Supplemental Figure 2A). UNC45A-KO cells were severely depleted of the typical NMII-associated filaments observed in parental U2OS cells. Instead, we found irregular and fragmented NMIIA bundles, mainly at the outer edges, and diffuse NMIIA staining in the cytoplasm of UNC45A-KO cells. The formation of numerous NMIIA-positive aggregates after proteasome inhibition with MG-132 further indicated that a large number of NMIIA molecules are not properly folded in UNC45A-KO cells (Figure 3B and Supplemental Figure 2A).

**Figure 4:**
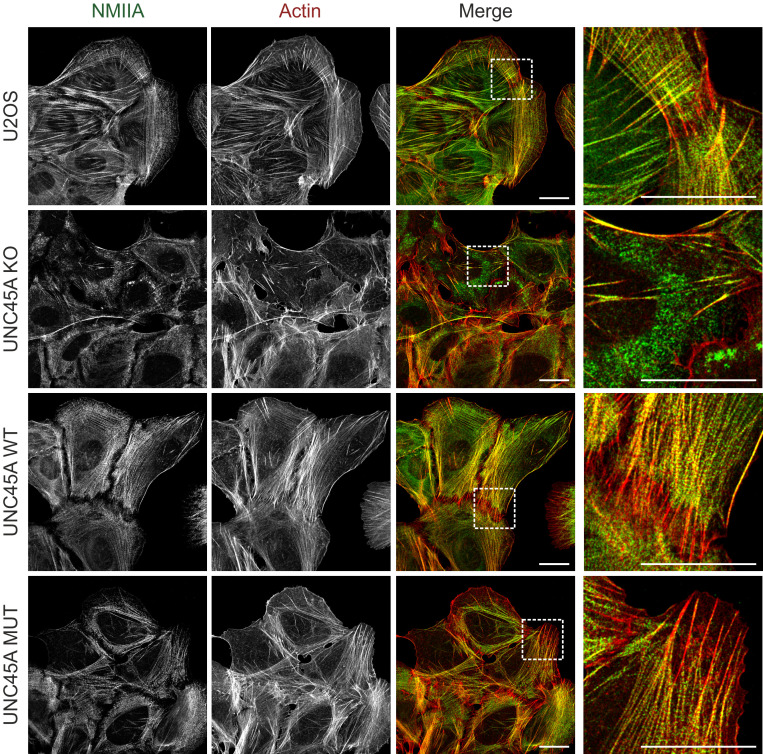
UNC45A p.Leu237Pro rescues stress fiber formation in U2OS UNC45A-KO cells. Confocal immunofluorescence microscopy images of U2OS, UNC45A-KO, and stably complemented UNC45A wild-type and mutant cells costained with markers for actin (red) and NMIIA (green). Stress fiber formation was severely affected in UNC45A-KO cells. Compared with this, UNC45A mutant rescue cells exhibited a regular actomyosin network similar to U2OS and UNC45A wild-type–expressing cells. Scale bars, 20 μm.

Actin filaments, the major component of stress fibers (31), were also abnormally distributed in U2OS UNC45A-KO cells. UNC45A deficiency caused ring-like phalloidin staining at the cell periphery, but the cytoplasmic network of actin filaments was mostly absent (Supplemental Figure 2B). The abnormal organization of NMIIA and actin filaments greatly reduced stress fiber formation in UNC45A-KO cells (Figure 4). Ectopic expression of wild-type UNC45A and the p.Leu237Pro mutant restored the organization of NMIIA and actin, as well as the stress fiber network, while preventing MG-132–induced aggregation of NMIIA in UNC45A-deficient cells (Figure 3B, Figure 4, and Supplemental Figure 2, A and B). Importantly, neither UNC45A deficiency nor the p.Leu237Pro variant affected microtubule organization in these cells (Supplemental Figure 3).

NMII critically regulates the assembly and maturation of focal adhesions by influencing the turnover of the focal adhesion–associated proteins vinculin, paxillin, and zyxin (32, 33). Consistent with impaired NMII functions, the formation of focal adhesions was strongly diminished in U2OS UNC45A-KO cells (ref. 18 and Supplemental Figure 4). Expression of either the wild-type or the mutant UNC45A protein restored focal adhesions that were positive for both paxillin and zyxin. Whereas paxillin primarily coordinates signaling pathways critical for focal adhesion dynamics and cell migration, zyxin is involved in actin assembly, mechanosensing, and stress response (34–36). Together, our results revealed that the UNC45A p.Leu237Pro mutant retained chaperone- and filament assembly–specific functions necessary for NMII folding and formation of NMII-actin cellular complexes.

### The p.Leu237Pro variant enforces oligomer formation and prevents dissociation of chaperone-myosin complexes.

**Figure 5:**
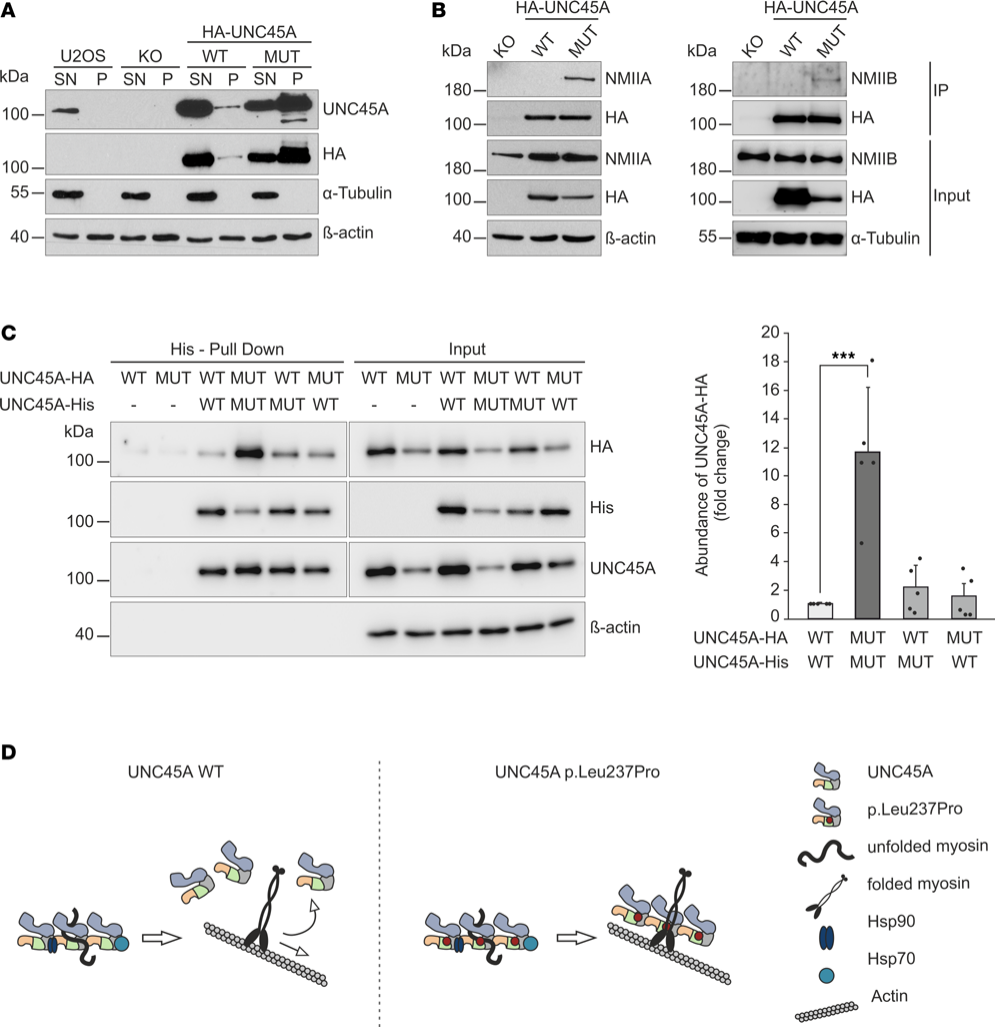
Oligomerization of the UNC45A p.Leu237Pro variant and complex formation with nonmuscle myosins. (**A**) Distribution of endogenous and ectopic HA-tagged UNC45A wild-type and mutant proteins in NP-40–soluble (SN) and –insoluble (P) fractions of U2OS, UNC45A-KO, and complemented cells was visualized by immunoblot using UNC45A- and HA-tag–specific antibodies. α-Tubulin and β-actin served as control for fractionation efficiency and protein loading, respectively. (**B**) HA-tagged UNC45A wild-type and mutant proteins were expressed in U2OS UNC45A-KO cells and co-immunoprecipitated with anti-HA antibody. Immunocomplexes were analyzed with specific antibodies directed against the HA-tag, NMIIA, and NMIIB to detect UNC45A and interacting myosins, respectively. α-Tubulin and β-actin were used as loading controls. (**C**) U2OS UNC45A-KO cells were cotransfected with His- and HA-tagged UNC45A wild-type and mutant constructs in various combinations. His-tagged proteins were pulled down with Ni-NTA Agarose beads, and precipitated complexes were analyzed for the presence of HA-tagged proteins. Amounts of HA- and His-tagged UNC45A and cell lysates (input) were analyzed using indicated antibodies. β-Actin served as a loading control for the input samples. Intensities of the immunoblot signals were quantified using ImageJ (Fiji) and are presented as a fold-change. Results from 5 independent experiments are shown. His- and HA-tagged UNC45A wild-type oligomers were taken as 1, and significance was scored with the Student’s *t* test. *** *P* < 0.001. (**D**) Graphical representation of the chaperone-myosin interference by UNC45A p.Leu237Pro mutant. UNC45A forms transient multimers assisting in binding and folding of fully functional myosins. In contrast, the p.Leu237Pro mutant forms atypically stable oligomers, thus remaining bound to myosin complexes and limiting myosin functions.

Detergent extraction experiments showed that a small amount of UNC45A localizes to NMII-containing actin stress fibers in a manner that binds myosin II. In addition, live-cell photoactivation experiments revealed a transient interaction between UNC45A and cytoskeletal actomyosin complexes characterized by rapid association and dissociation rates (18). We analyzed the distribution of wild-type UNC45A and the p.Leu237Pro mutant in the NP-40 detergent–soluble (SN) and cytoskeleton-enriched detergent-insoluble (P) fractions of U2OS cells. Consistent with previous findings, the majority of endogenous and ectopic wild-type UNC45A proteins were found in the NP-40–soluble fraction with only a small portion present in the detergent-resistant pellets (Figure 5A). However, the distribution of the mutant chaperone differed from that of the wild-type protein. The p.Leu237Pro variant was predominantly found in the insoluble fraction, suggesting an increased propensity to associate with cytoskeletal proteins such as NMII.

To validate this hypothesis, we immunoprecipitated wild-type or p.Leu237Pro UNC45A proteins expressed in U2OS UNC45A-KO cells and analyzed for nonmuscle myosin proteins in the immune complexes. NMIIA and NMIIB could not be detected in the wild-type UNC45A-specific complexes (Figure 5B), verifying the transient nature of the chaperone-myosin interactions as reported in previous work (8, 18). Notably, we found that both NMII forms were co-immunoprecipitated with p.Leu237Pro UNC45A, indicating that these proteins formed stable cellular complexes. This could explain the increased association of the p.Leu237Pro protein with the cytoskeleton shown by cell lysate fractionation (Figure 5A).

Previous studies have shown that *Caenorhabditis elegans* UNC-45 (ceUNC-45) forms a linear homo-oligomeric chain that serves as an ideal docking platform with numerous myosin-binding sites. This structural arrangement facilitates efficient myosin interactions (8). Furthermore, higher-order ceUNC-45 oligomer formation via the TPR domain is critical to setting up an induced-fit myosin-binding UCS canyon, thus linking UNC-45 oligomerization to its myosin binding and folding activities (8, 9). Although such studies have not been performed for UNC45A, it is thought that similar mechanisms may apply to this highly homologous chaperone form. Here, we established a nickel-nitrilotriacetic acid–based (Ni-NTA–based) pull-down assay that allowed us to investigate the oligomerization of UNC45A in cells. His- and HA-tagged wild-type and p.Leu237Pro UNC45A proteins were expressed in various combinations in U2OS UNC45A-KO cells. His-tagged UNC45A–specific protein complexes were isolated using Ni-NTA Agarose. As shown in Figure 5C, HA-tagged UNC45A was specifically pulled down with His-tagged UNC45A bait protein, indicating complex formation between these proteins. When the p.Leu237Pro mutant was used in this assay, we observed a strong increase in the retention of HA-tagged UNC45A prey protein. We conclude that the p.Leu237Pro variant abnormally promotes the stability of UNC45A-specific oligomers, which normally exist as short-lived protein complexes (8). Interestingly, when we coexpressed the p.Leu237Pro mutant protein with the wild-type protein, normal levels of UNC45A complexes were restored, indicating a recessive effect of this mutation (Figure 5C). Taken together, these results reveal an abnormal accumulation of p.Leu237Pro UNC45A in the cytoskeleton, consistent with enhanced oligomer formation and stabilization of chaperone-NMII interactions (Figure 5D).

### The UNC45A p.Leu237Pro variant alters Tfn recycling through NMII substrate interaction.

NMII and UNC45A have been identified in a genome-wide siRNA screen as essential factors involved in clathrin-mediated endocytosis (21). Actin-NMII complexes interact with a range of endocytic vesicles and various Rab GTPases, facilitating the morphogenesis of endomembrane compartments and supporting fission events (37–39). These mechanisms are central to the regulation of endosomal trafficking and recycling and help maintain the balance of sorting compartments. Consistent with this, experimental inhibition of NMII function has been shown to disrupt endocytic trafficking and impair recycling of Tfn (37, 39).

We investigated whether the abnormally stable association of actomyosin complexes with the UNC45A p.Leu237Pro variant disturbed their specific roles in endocytic processes. To this end, we investigated Tfn uptake and recycling in UNC45A-KO and reconstituted U2OS cell lines, as well as in control cells. First, we used a pulse-chase approach combined with cold conditions to follow the early stages of Tfn internalization (Supplemental Figure 5). Serum-starved cells were treated with Tfn conjugated to Alexa Fluor 488 on ice to prevent internalization, followed by a shift to 37°C to allow uptake. We observed that at time 0, Tfn–Alexa Fluor 488 was bound to the cell surface and rapidly internalized in all U2OS cell lines, manifesting as small punctate structures in the cytoplasm. By 10 to 20 minutes, Tfn had also accumulated in the perinuclear region in all genotypes. These results suggest that UNC45A is not critical for the early steps of transferrin endocytosis.

**Figure 6:**
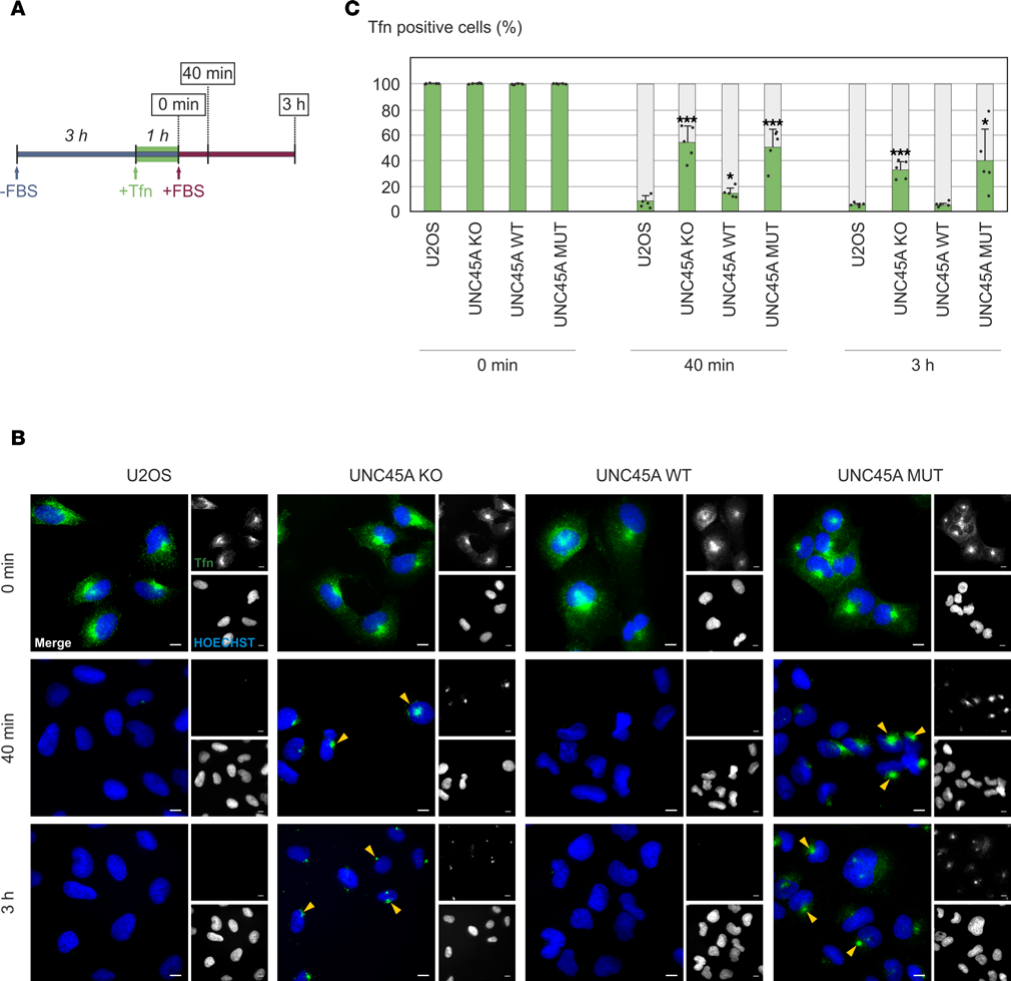
Epifluorescence microscopy of impaired Tfn recycling in UNC45A-KO and p.Leu237Pro mutant U2OS cells. (**A**) Schematic representation of a Tfn recycling assay as detailed in Methods. (**B**) U2OS, UNC45A-KO, as well as UNC45A wild-type and mutant rescue cells were treated with Tfn–Alexa Fluor 488 (green) and allowed to recycle for different durations. Nuclei were stained with HOECHST (blue). The single channels for both Tfn signal and nuclei are right from the merged images. Perinuclear Tfn clusters in UNC45A-KO and p.Leu237Pro mutant cells persist even after prolonged recycling (yellow arrowheads). Scale bars, 10 μm. (**C**) Quantification of Tfn-positive cells at different time points of the Tfn recycling assay by using ImageJ. Five independent fields were scored, and statistical analysis was made with the Student’s *t* test. * *P* < 0.05, *** *P* < 0.001.

Transferrin recycling was next examined. Serum-starved cells were incubated with Tfn–Alexa Fluor 488 for 1 hour at 37°C to achieve intracellular saturation. After removal of excess Tfn–Alexa Fluor 488, cells were either fixed immediately or returned to complete medium for different periods (Figure 6A), during which the subcellular distribution of Tfn was recorded. At the initial time point, control, UNC45A-KO, and p.Leu237Pro mutant U2OS cells all exhibited similar distributions and fluorescence intensities of Tfn–Alexa Fluor 488–positive compartments (Figure 6, B and C). After washout and a 40-minute recovery period, Tfn–Alexa Fluor 488 was no longer detectable in the majority of parental and UNC45A WT cells. In contrast, the majority of UNC45A-KO and p.Leu237Pro mutant cells continued to show Tfn accumulation in the perinuclear area, and even after 3 hours of recovery, nearly half of these cells retained Tfn (Figure 6, B and C). Incorporation of the lysosomal inhibitor leupeptin into the incubation protocol produced nearly identical patterns, effectively ruling out catabolic activity as a reason for the loss of Tfn fluorescence (Supplemental Figure 6). This evidence suggests that Tfn recycling is impaired in UNC45A-KO and p.Leu237Pro mutant cells, whereas this impairment can be reversed in UNC45A WT cells.

**Figure 7:**
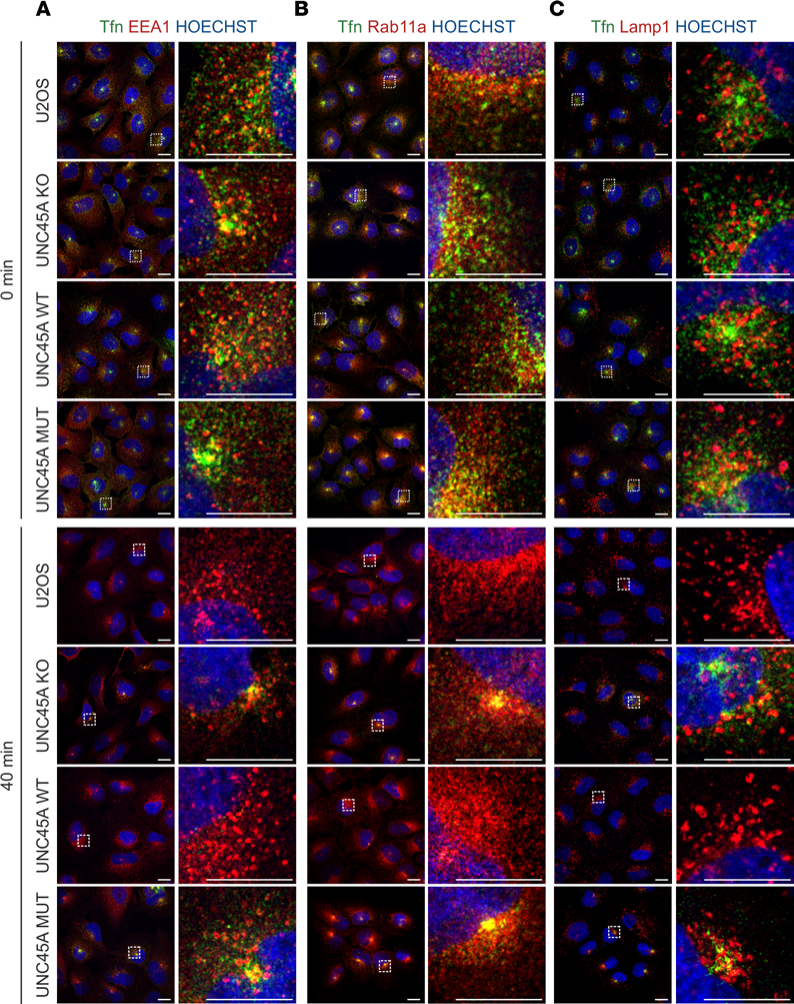
Confocal IF characterization of Tfn-containing endocytic compartments in U2OS cell lines. U2OS parental, UNC45A-KO, and stably complemented UNC45A wild-type and mutant cells were subjected to the Tfn recycling assay as described in Figure 6A and Methods. The cells were fixed immediately after Tfn saturation (time point 0 minutes) and after 40 minutes of recycling. Cells were labeled with markers for (**A**) early/sorting endosomes (anti-EEA1), (**B**) recycling endosomes (anti-Rab11a), and (**C**) late endosomes (anti-Lamp1). Nuclei were stained with HOECHST (blue). The samples were analyzed using the LSM980 AiryScan2 confocal microscope (Zeiss). In all genotypes investigated similar Tfn distribution was seen after Tfn saturation, but only UNC45A-KO and p.Leu237Pro mutant cells showed persistent Tfn accumulation in early/sorting endosomes, recycling endosomes, and to a certain extent also late endosomes after 40 minutes of recycling. Scale bars, 20 μm. EEA1, early endosome antigen 1; Rab11a, Ras-related protein Rab-11A; LAMP1, lysosome-associated membrane protein 1.

**Figure 8:**
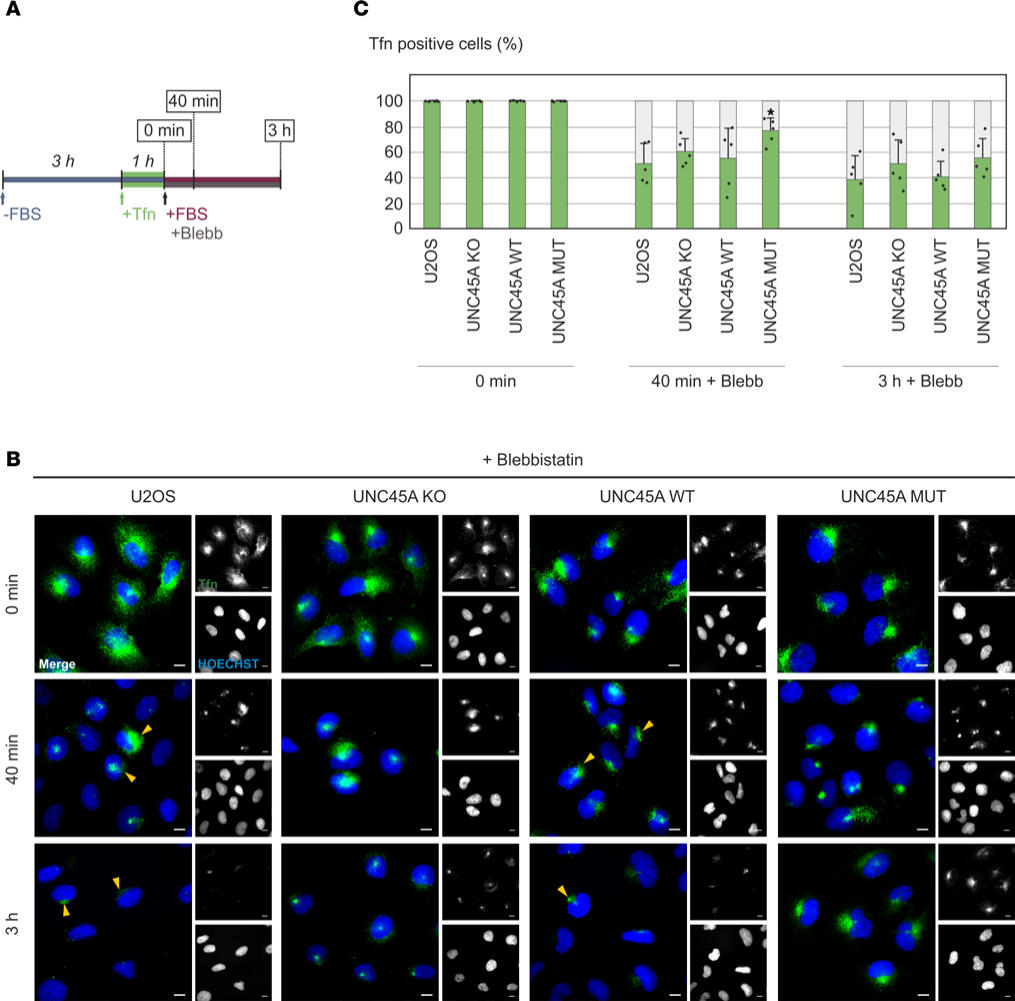
Pharmacological inhibition of NMII in U2OS control and UNC45A-KO wild-type rescue cells mimics the Tfn recycling defect of UNC45A-KO and p.Leu237Pro mutant cells. (**A**) Scheme of the Tfn recycling assay in the presence of para-aminoblebbistatin (Blebb). (**B**) Representative images of U2OS cells with internalized Tfn–Alexa Fluor 488 in green and nuclei in blue. Para-aminoblebbistatin (Blebb) was present throughout the recycling phase in a concentration of 50 μM, and Blebb-induced perinuclear Tfn accumulation in control and wild-type rescue cells is indicated by yellow arrowheads. Scale bars, 10 μm. (**C**) Quantification of Tfn-positive cells. Data were analyzed as described in Figure 6C. * *P* < 0.05.

This observation was further supported by an initial characterization of Tfn-positive compartments by IF confocal microscopy (Figure 7, A–C, and Supplemental Figures 7 and 8). After 1 hour of internalization, Tfn–Alexa Fluor 488 colocalized with EEA1-positive early/sorting endosomes and Rab11a-positive recycling endosomes in all genotypes. However, there was less colocalization with Lamp1-positive endolysosomal compartments. After washout and subsequent 40-minute incubation in complete culture medium, only UNC45A-KO and p.Leu237Pro mutant cells continued to show distinct Tfn fluorescence within early/sorting endosomes and recycling endosomes. Notably, at this time point, these cells also exhibited some residual Tfn–Alexa Fluor 488 in endolysosomal compartments, reminiscent of the Tfn misdirection and jamming previously reported under NMII-inhibitory conditions (39). These results highlight the disruption of both the fast and slow Tfn recycling pathways (40, 41) in these mutant and UNC45A-deficient cells. Previous studies have documented disturbances in Tfn endocytosis and recycling following inhibition, depletion, or knockout of NMII (37, 39). With this in mind, we examined Tfn recycling in our U2OS cells treated with blebbistatin, a specific inhibitor of NMII ATPase activity and assembly (42). Our results showed that blebbistatin effectively blocked the release of Tfn–Alexa Fluor 488 from perinuclear clusters in both parental and UNC45A WT U2OS cells, a phenotype that closely mirrors the recycling defect observed in UNC45A-KO and p.Leu237Pro mutant cells (Figure 8, A–C). The inclusion of leupeptin in these experiments demonstrated that lysosomal degradation was not a contributing factor (Supplemental Figure 9). Together, these results strongly suggest that the UNC45A p.Leu237Pro variant disturbs Tfn recycling by interfering with NMII-dependent mechanisms, representing a dysfunction similar to that seen in UNC45A deficiency. This underscores the critical roles of both UNC45A and NMII in the proper functioning of Tfn recycling pathways.

### Genetically engineered CaCo2 cell models mimic histopathology of enterocytes in patient P1.1 with UNC45A c.710T>C missense variant.

One of the most important facets of O2HE is severe congenital secretory diarrhea (1). We previously reported small intestinal biopsies from P1.1 carrying the missense variant c.710T>C (p.Leu237Pro) to present the histological hallmarks of MVID-like enteropathy (5). To provide further experimental evidence in vitro that the c.710T>C (p.Leu237Pro) variant is causally related to the observed intestinal histopathology, we extended our microscopic studies to more disease-relevant cell models derived from the enterocyte-like CaCo2 cell line. CaCo2 UNC45A-KO cells, as well as CaCo2-KO cells genetically engineered with the c.710T>C (p.Leu237Pro) variant, should recapitulate the enterocyte phenotype observed in the patient. In contrast, restoration of wild-type UNC45A should correct these defects (Supplemental Figure 10A). Suitable clones from stable CaCo2 cell lines, plus parental CaCo2 controls, were cultured for more than 21 days on semipermeable filter membranes. The resulting polarized and differentiated epithelial cell monolayers were thoroughly analyzed by cryo-based transmission and scanning electron microscopy (Supplemental Figures 10–12). The patterns of p.Leu237Pro variant and UNC45A-KO cells displayed the full range of ultrastructural abnormalities of MVID-like enteropathy similar to P1.1’s enterocytes (Supplemental Figure 10B and Supplemental Figure 11B). All those alterations could be reverted by genetic reconstitution with wild-type UNC45A (Supplemental Figure 11, A and B). Unlike parental WT CaCo2 cells (Supplemental Figure 11, A and B), apical microvilli in UNC45A-deficient and mutant cells were heterogeneous in size and shape and appeared irregular, and cells lacked a dense, continuous brush border (Supplemental Figure 10B and Supplemental Figure 11B). Ectopic microvilli manifested as characteristic “microvillous inclusions” or as basolateral assemblies, each with different levels of complexity. In addition, (autophago)lysosomes appeared to be somewhat enlarged (Supplemental Figure 10B). Finally, prominent tubulovesicular compartments were frequently observed beneath both native and ectopic apical surfaces of UNC45A-deficient and p.Leu237Pro mutant cells (Supplemental Figure 10B). When analyzed by immunogold labeling electron microscopy, these compartments stained positive for Rab11a (Supplemental Figure 12A). Enterocytes from MVID patients with mutations in MYO5B, STX3, or STXBP2 show similar structures (43–45). Several lines of evidence suggest that these subapical endomembranes are indeed recognized as recycling endosomes (43), though their irregular distribution differs from the typical location found in healthy polarized epithelial cells (46). These structures make up the majority of the subapical periodic acid–Schiff–positive (PAS-positive) organelles specific for MVID, which have historically been referred to as “secretory granules” (47, 48).

Immunoelectron microscopy was used to expand previously published immunofluorescence data (5) on 2 disease-relevant transporters: dipeptidyl peptidase-4 (DPPIV), which is correctly positioned, and sodium–hydrogen exchanger 3 (NHE3), which is partially mislocalized. Similar to findings in patient biopsies (5), both transporters were localized to the brush border of all our CaCo2 genotypes (Supplemental Figure 11C and Supplemental Figure 12B). However, it is important to note that determining the relative intensity of brush border labeling is challenging because of the mosaic expression patterns observed in individual monolayers (49), and this analysis was beyond the scope of our study. Of interest, however, seems the fact that NHE3 immunogold label associated in UNC45A-KO and p.Leu237Pro cells additionally with mainly vesicular compartments (Supplemental Figure 12B) located in the subapical cytoplasm, as did Rab11a. This is consistent with findings indicating either a normal distribution (50) or partially altered localization (43–45) of these transporters at the brush border and (recycling) endosomes, which varies according to specific physiological or pathological conditions, especially in cases of MVID-like enteropathies.

In conclusion, our CaCo2 models provide a reliable in vitro representation of the ultrastructural characteristics of duodenal enterocytes derived from patient biopsies, particularly with respect to the subcellular distribution of 2 transporters essential for intestinal physiology. Importantly, all alterations observed in UNC45-KO and mutant cells could be reversed by genetic reconstitution with wild-type UNC45A (Supplemental Figure 11, A and B, and Supplemental Figure 12, A and B). This reinforces the critical role of UNC45A and its p.Leu237Pro variant in the pathophysiology of MVID-like disorders and underscores the efficacy of the CaCo2 model in studying these underlying mechanisms.

## Discussion

Loss-of-function mutations in the myosin cochaperone UNC45A lead to the onset of O2HE syndrome in newborns, characterized by manifestation of all or a number of these symptoms: severe diarrhea, cholestasis, deafness, bone fragility, and mild intellectual disability (1). Previously, we have shown that UNC45A is required to maintain epithelial cell polarity and apicobasal trafficking by promoting MYO5B folding and that UNC45A deficiency is associated with an MVID-like intestinal phenotype in patients with O2HE (5). Pathogenic variants have been identified in all domains of UNC45A, generally resulting in loss of the protein or its specific functions.

Here, we studied the *UNC45A* c.710T>C (p.Leu237Pro) missense variant, which was found as a homozygous or compound heterozygous mutation in 4 newborns from 3 nonrelated families (5, 22). All patients had life-threatening diarrhea and cholestasis with or without bone fragility, symptoms associated with O2HE (1). All cases showed severe disease progression requiring parenteral nutrition therapy. Of note, 1 patient underwent a complete enterectomy, whereas 2 cases resulted in early mortality. A Chinese neonate reported by Kong et al. showed additional complications of the heart and brain (22), broadening the phenotypic spectrum of the p.Leu237Pro variant. Despite its associated severe phenotype, the UNC45A p.Leu237Pro was expressed at wild-type levels and retained chaperone activity sufficient for myosin folding and actomyosin filament assembly, suggesting a complex and distinct pathogenic mechanism.

To characterize the UNC45A p.Leu237Pro variant, we employed different experimental systems. Fibroblasts obtained from skin biopsies of homozygous and heterozygous carriers were used to compare the endogenous expression levels of wild-type and p.Leu237Pro UNC45A, together with its specific substrate, NMIIA, in both a patient and a healthy individual. Our results show that the p.Leu237Pro variant does not affect the expression of UNC45A and NMIIA proteins in cells from homozygous patients. It also has no dominant-negative effect on wild-type allele function in heterozygous individuals.

Building on the role of UNC45A in NMII folding and filament formation in U2OS cells (18), we complemented U2OS UNC45A-KO cells with the p.Leu237Pro construct to investigate the effect of the variant. Since these cells lack UNC45B (18), we could investigate the impact of the UNC45A p.Leu237Pro variant and the underlying mechanism, excluding potential compensatory effects of this other chaperone isoform. Intriguingly, the expression, protein stability, and myosin folding activity of the mutant were also comparable to wild-type UNC45A in U2OS cells. Like wild-type UNC45A, the mutant effectively restored NMIIA and MYO5B expression, as well as NMIIA and actin organization. It also supported the assembly of actomyosin-specific structures, such as stress fibers and focal adhesions, in complemented U2OS UNC45A-KO cells. These findings suggest that the pathogenicity of this variant is different from other O2HE-specific UNC45A mutations linked to chaperone destabilization or inactivation, even though it is associated with a severe phenotype in patients. Further examination revealed an unusual enrichment of the p.Leu237Pro mutant in the detergent-insoluble cytoskeletal fraction of U2OS cells caused by the abnormal increase in formation of UNC45A complexes with NMII. Most molecular chaperones act bidirectionally, with the highest affinity for the unfolded state and the lowest affinity for the native state of their substrates, to prevent sequestering of correctly folded proteins (13). Similarly, UNC45A showed a transient interaction with NMII-containing stress fibers, as defined by rapid association and dissociation dynamics in photoactivation studies, while a stable association was only seen with aggregates composed of misfolded myosin (18). The UNC45A p.Leu237Pro variant displayed unique behavior, remaining associated with NMIIA- and NMIIB-specific complexes despite its chaperone activity and role in preventing myosin aggregation. We assumed that the pathogenic effects of the mutation were caused by altered chaperone interactions rather than a loss of chaperone function.

Previous studies showed that binding of the smooth muscle UNC45B isoform to myosin heads inhibited the myosin power strokes without affecting myosin-specific ATPase activity. The findings suggest that the central domain of UNC45B induces conformational changes in the myosin-UNC45B complex through allosteric interactions, stabilizing an inactive actomyosin conformation and blocking motor translocation (51–53). Based on the high protein homology between UNC45 isoforms — approximately 56% identity and 74% similarity in their amino acid sequences — as well as predicted structural similarities, we hypothesize that the central domain of UNC45A, similar to UNC45B, may interact with NMII and inhibit its actin translocation function. In this context, stable binding of the UNC45A p.Leu237Pro mutant to myosin could disrupt its actin sliding activity and actomyosin-specific functions in nonmuscle cells. This interaction between the chaperone and myosins could explain the loss-of-function phenotype observed in patients with O2HE syndrome.

CeUNC-45 has been shown to form transient multimers of 2 to 5 subunits in vitro, acting as a myosin docking platform with multiple binding sites. The proposed multimeric model suggests that a relatively small dimerization interface in the TPR and neck domains facilitates the rapid assembly and disassembly of these oligomers. The mobility of the chaperone within sarcomere structures depends on this dynamic (8). Oligomer-deficient mutants of ceUNC-45 tend to adopt a curved conformation of the UCS domain, restricting functional myosin binding, and fail to rescue muscle sarcomere organization in worms with a temperature-sensitive unc-45 allele (8, 11). We have shown that UNC45A can dimerize in vivo to form cellular homo-oligomeric UNC45A complexes. Notably, the p.Leu237Pro variant significantly enhances UNC45A oligomerization, likely by increasing structural flexibility and promoting a conformation conducive to stable oligomers. This enhanced oligomerization may explain the increased myosin binding observed with the UNC45A p.Leu237Pro variant, which inhibits myosin activity by preventing its dissociation from chaperone-myosin complexes. Coexpression of wild-type UNC45A effectively reduced dimerization of UNC45A subunits, thereby neutralizing the effect of the p.Leu237Pro variant and preventing dominant-negative effects. This is consistent with the healthy status of the heterozygous carriers and explains the recessive nature of the *UNC45A* c.710T>C variant.

The proposed mechanism of the UNC45A p.Leu237Pro variant allows us to differentiate the effects of UNC45A deficiency from those caused by the mutated chaperone. In the absence of UNC45A, myosin does not reach a functional folded state and is unable to assemble actomyosin complexes, which leads to disruption of NMII-dependent cellular processes. Conversely, the mutant chaperone p.Leu237Pro not only binds and folds myosins but also forms unusually stable oligomers, thus remaining attached to myosin complexes and limiting myosin functionality (Figure 5D). We propose that pharmacological targeting of the chaperone-myosin interaction or dimerization of UNC45A p.Leu237Pro may pose as a potential therapeutic option for this patient population.

Intestinal features of the 2 histologically described cases included — among others — subapical accumulation of misdirected cargo (5). Similar to MVID caused by MYO5B, STX3, or STXBP2 mutations (43, 44), this traffic blockade resulted from impairment of MYO5B-Rab11/Rab8–mediated pathways through apical recycling compartments. Our study on U2OS provides direct evidence that Tfn recycling, another classical cargo-recycling process, depends in those nonpolarized cells on the correct interaction of the UNC45A chaperone with NMII. We were able to show that the loss of UNC45A, the expression of the p.Leu237Pro variant, or the pharmacological suppression of NMII impaired the recycling of Tfn and led to abnormal cargo retention in different endocytic compartments. The cargo accumulated in EEA1-positive early/sorting endosomes and Rab11a-associated vesicles that represent different stages of Tfn recycling (54). Thus, our findings provide further evidence that the UNC45A chaperone is an essential factor in intracellular trafficking and cargo sorting by regulating NMII in addition to MYO5B. Altered distribution of the Tfn receptor has been observed in intestinal epithelia from certain patients with MVID (19), but Tfn recycling defects have, to our knowledge, not been reported in any MVID cell model. However, the actual contribution of NMII and Rab11a-MYO5B complexes to the observed alterations in Tfn recycling in UNC45A-deficient and p.Leu237Pro mutant U2OS cells requires more investigation.

The intestinal phenotype associated with severe secretory diarrhea observed in patients with O2HE is caused by brush border atrophy and mislocalization of channels and transporters essential for nutrient absorption and homeostasis (5). The establishment and maintenance of polarity, and thus the integrity of enterocytes, is mediated by different classes of myosins. For example, the contractility of NMII-dependent actin filament turnover regulates the length of epithelial microvilli; inhibition of this process results in brush border disorganization, microvilli elongation, and decreased retrograde flow (55). NMII is also involved in forming and maintaining apical junctions that control epithelial barrier permeability and function (56, 57). In addition, NMII plays a critical role in fission events that are essential for both the early stages of transport, when new vesicles form and detach from a donor compartment, and the later stages, when cargo vesicles are delivered to their specific destinations (37, 39, 58). In complex with Rab11 and Rab8 GTPases, MYO5B mediates transport of a selected cargos acting through apical recycling compartments (59–61) whereas actin-bound class I myosins contribute primarily to brush border organization (20, 62, 63), cell-cell adhesion (64), and the maintenance of anterograde and retrograde transport routes (65). Since these myosins are substrates of UNC45A (5, 18), it is important to study the effects of abnormal UNC45A binding and chaperone-myosin perturbations on enterocyte-specific functions. This will deepen our insight into MVID-related enteropathies and help develop more effective therapeutic strategies.

In conclusion, our study provides a thorough molecular and functional analysis of the *UNC45A* c.710T>C variant and highlights its distinct pathogenic features. Further research is essential to improve our understanding of how UNC45A-related mechanisms contribute to the complex symptoms of O2HE syndrome.

## Methods

### Sex as a biological variable.

The sex was not considered as a biological variable in this study.

### Patient information and molecular genetic studies.

An overview of patients and their main clinical manifestations is provided in Table 1. For more detailed patient descriptions, see the respective publications (5, 22). Skin biopsies from members of family 2 and healthy volunteers were obtained for diagnosis and further investigation. Genetic analyses including next-generation and conventional Sanger sequencing were performed on DNA extracted from peripheral mononuclear cells as previously described (66). Exome sequencing was performed in patient P2.1 as described (67). Briefly, Agilent’s Sure-SelectXT2 V6 enrichment kit and an Illumina HiSeq 4000 instrument were used to generate 150 bp paired-end reads that were aligned to the human reference genome hg19 with Burrows-Wheeler transformation (68). PCR duplicates were removed with PICARD (http://broadinstitute.github.io/picard/) and single nucleotide substitutions, and small indels were called with SAMtools software. All variants were submitted to SeattleSeq (https://snp.gs.washington.edu/SeattleSeqAnnotation/; service is no longer available) for annotation, categorization, and filtering against public variant databases. The UNC45A variant designation is based on the NCBI reference sequence for transcript NM_018671.5 (corresponding to Ensembl transcript reference sequence ENST00000418476.2) and the genomic reference sequence NG_061633.1 (corresponding to Ensembl gene ENSG00000140553). Nucleotide numbering uses + 1 as the A of the ATG translation initiation codon in the reference sequence, with the initiation codon as codon 1.

### Cell culture and cell lines.

U2OS and U2OS UNC45A-KO cells, generated by CRISPR/Cas9 technology, were provided by Pekka Lappalainen (Institute of Biotechnology, University of Helsinki, Helsinki, Finland) and characterized before (18). Enterocyte-like CaCo2 cells were obtained from the American Type Culture Collection (HTB-37, 60143947), and the UNC45A-KO cell line was established as described previously (5). Primary fibroblasts were established from skin biopsies by standard procedure. U2OS cells, CaCo2 cells, or fibroblasts were cultured in DMEM (Thermo Fisher Scientific, 31966-021) supplemented with either 10% or 20% fetal bovine serum (FBS; MilliporeSigma, S0615), respectively, and 100 U/mL penicillin and 100 μg/mL streptomycin (MilliporeSigma, P0781) at 37°C in a humidified atmosphere with 5% CO_2_.

### Antibodies and reagents.

The following primary antibodies were used for immunoblotting: anti-UNC45A (ENZO, ADI-SRA-1800-F; and Invitrogen, PA5-58703, both in a dilution of 1:1,000), 6x-His-tag monoclonal antibody (Invitrogen, MA1-21315, diluted 1:1,000), anti-HA (BioLegend, 101501, diluted 1:1,000), anti-p53 (Santa Cruz Biotechnology, sc-126, diluted 1:1,000), anti-MYO5B (Novus Biologicals, Bio-Techne; NBP1-87746, diluted 1:500), and anti–β-actin (Cell Signaling Technology, 3700S, diluted 1:1,000). For immunoblotting, anti-NMIIA (BioLegend, 909801), anti-NMIIB (BioLegend, 909901), and anti–α-tubulin (DSHB, 12G10) were used in dilutions of 1:1,000 and for IF at 1:500. Primary antibodies for IF included anti-EEA1 (Santa Cruz Biotechnology, sc-33585) and anti-Rab11a (Cell Signaling Technology, 2413S), in dilutions of 1:100 and 1:50, respectively; anti-LAMP1 (BD Pharmingen, 553792) diluted 1:500; anti-Paxillin (BD Transduction Laboratories, 610051) diluted 1:100; anti-Zyxin (Proteintech, 10330-1-AP) diluted 1:50; and anti-HA (Cell Signaling Technology, 3724S) diluted 1:1,000. Alexa Fluor 568–conjugated phalloidin (Invitrogen, A12380) was used in a dilution of 1:500 to visualize the actin cytoskeleton, and nuclei were stained with HOECHST (MilliporeSigma, B2261) diluted 1:10,000. Secondaries for immunoblotting were horseradish peroxidase–conjugated goat anti-mouse IgG (MilliporeSigma, A4416) and goat anti-rabbit IgG (MilliporeSigma, A0545), both in a dilution of 1:4,000. For IF, we used secondary goat anti-mouse IgG Alexa Fluor 488 and 568 (Invitrogen, A-11001 and A-11031, respectively) and goat anti-rabbit IgG Alexa Fluor 488 and 568 (Invitrogen, A-11008 and A-11011, respectively), all in a dilution of 1:1,000.

### UNC45A protein structure modeling.

The 3D protein model of human UNC45A from AlphaFold (AFBD: AF-Q9H3U1-F1) (69) served as a template for further structural analyses. Vibrational entropy changes between wild-type and mutant UNC45A were calculated using the DynaMut web server (23). Noncovalent molecular interactions in the model were determined using Arpeggio (70), and the change in free energy was predicted with the ENCoM method (24).

### Construction of cDNA clones.

The UNC45A fragment was PCR-amplified from human cDNA with primers for nontagged, N-terminal HA-tagged, and N-terminal 6x-His–tagged versions, which were subsequently cloned into the pcDNA3.1 (+) expression vector. The *UNC45A* c.710T>C point mutation was introduced by site-directed mutagenesis. To generate UNC45A-expressing viral constructs, the nontagged versions of wild-type and mutant UNC45A were cloned into the BamHI and SalI restriction sites of a pRRL vector containing an elongation factor-1 alpha (EFS-NS) promotor. A cap-independent translation enhancer fused to the puromycin resistance gene and the woodchuck hepatitis virus posttranscriptional regulatory element were introduced downstream of the UNC45A coding sequence. For the complementation of CaCo2 UNC45A-KO cells, the resistance gene was replaced by the blasticidin resistance gene. All generated constructs were sequence verified.

### Stable expression of wild-type and mutant UNC45A in U2OS and CaCo2 UNC45A-KO cells.

To complement U2OS and CaCo2 UNC45A-KO cells with UNC45A wild-type and mutant constructs, lentiviral particles were prepared. HEK293T cells (ATCC) were transfected with the respective transfer plasmids, packaging plasmids psPAX2, and VSV-G envelope plasmid pMD.G using the calcium phosphate coprecipitation method (66). Viral supernatants were harvested and concentrated with the Retro-X Concentrator (Clontech, Takara Bio USA Inc., 631455) 48 hours after transfection and used to transduce U2OS and CaCo2 UNC45A-KO cells in the presence of 4 μg/mL polybrene (MilliporeSigma, H9268-5G). Selection was performed with 5 μg/mL puromycin (Thermo Fisher Scientific, A1113803) for U2OS cells and 20 μg/mL blasticidin S (Gibco, A1113903) for CaCo2 cells. Single-cell clones with comparable UNC45A expression were chosen for further experiments.

### Transient expression of wild-type and mutant UNC45A proteins in U2OS UNC45A-KO cells.

For transient expression, U2OS UNC45A-KO cells were transfected with N-terminal HA-tagged or N-terminal 6x-His–tagged wild-type and mutant UNC45A using the calcium phosphate coprecipitation method (28). Five hours after transfection, the culture medium was replaced to a fresh one, and the cells were grown for 24–36 hours prior to analysis.

### Detergent fractionation and immunoblotting.

To analyze the distribution of UNC45A in detergent-soluble and -insoluble fractions, U2OS, UNC45A-KO, and transiently complemented cells were lysed using EB4 lysis buffer (40 mM Tris-HCl pH 8.0, 100 mM NaCl, 0.5% NP-40, 10 mM β-glycerophosphate, 10 mM NaF, 1 mM EDTA, 1 mM PMSF, and 1 μg/mL each of pepstatin, aprotinin, and leupeptin). The cell lysates were centrifuged at 16,000*g* at 4°C for 30 minutes. Supernatants, representing the detergent-soluble fraction, were collected and boiled in 1× SDS-PAGE sample buffer (40 mM Tris-HCl pH 6.8, 5% glycerol, 1% SDS, 100 mM DTT, 0.0025% bromophenol blue) at 95°C for 5 minutes. The detergent-resistant pellets were washed twice with the lysis buffer, solubilized in SDS-PAGE sample buffer, sonicated, and boiled at 95°C. For normalization, protein concentration was determined in supernatants by using the Coomassie Plus Protein Assay Reagent (Thermo Fisher Scientific, 1856210). Samples were separated by SDS-PAGE, transferred to nitrocellulose membrane (Amersham Protran Premium 0.2 NC, Cytiva 10600004), blocked with 5% milk in TBS-T (20 mM Tris-HCl pH 7.6, 137 mM NaCl, 0.05% Tween-20), and immunoprobed with indicated antibodies. Enhanced chemiluminescence (ECL) detection was carried out with commercial ECL solution (WesternBright Chemiluminescence Substrate Quantum, Biozym 541005), and the signal was visualized with the Fusion FX imaging system (Vilber) or using x-ray films.

### CHX chase assay.

U2OS UNC45A-KO cells were transiently transfected with wild-type and mutant UNC45A and left untreated or treated with 100 μg/mL CHX (MilliporeSigma, C7698-5G). Cells were harvested at the indicated time points and analyzed by immunoblotting.

### Thermal shift assay.

For the cell lysate thermal shift assay, U2OS UNC45A-KO cells were transiently transfected with HA-tagged UNC45A wild-type and mutant constructs. Cells were lysed in EB4 buffer and centrifuged at 16,000*g* for 25 minutes at 4°C. Supernatants were normalized and transferred to new tubes, and 0.01% SDS was added to prevent refolding of the proteins after heat denaturation. The lysates were divided into 20 μL aliquots and heated individually at different temperatures for 5 minutes (Thermocycler UNO96G Gradient, VWR) followed by cooling at room temperature. The samples were centrifuged at 16,000*g* for 30 minutes to pellet protein aggregates, spotted on a nitrocellulose membrane, and immunoblotted with anti-HA antibody. The ECL signal was visualized with the Fusion FX imaging system (Vilber), and the intensity of the detected dots was analyzed using ImageJ version vi.54f (Fiji) (71). Quantification of the data was performed using R. To estimate T_agg_, the nonlinear regression model 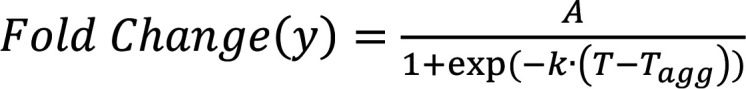, where A is the amplitude, exp is exponential function, k is the rate constant, T_agg_ is the aggregation temperature, and y is the fold-change, was used. This model was integrated into R as an object, and the package minpack.lm to implement fitting was applied. Starting points of our model were set at A = 1, k = 0.1, and T_agg_ = 50 for both the wild-type and the mutant form of the protein analyzed. The fold-change at each temperature was calculated by comparing each readout to the data point at 37°C. This was done independently for each replicate and each version of the protein, and the fold-change mean was used as the input for model fitting. The line in the figure (Supplemental Figure 1) illustrates the line of best fit, while the distribution of wild-type and mutant samples at specific temperature points provides context for the data within the framework of our model.

### Co-immunoprecipitation and oligomerization assay.

For co-immunoprecipitation, U2OS UNC45A-KO and transiently transfected cells were lysed in EDTA-free EB4 lysis buffer. Lysates were centrifuged at 16,000*g* for 20 minutes and precleaned with Protein A-Sepharose CL-4B beads (GE Healthcare, now Cytiva, GE17-0780-01) by rotating for 15 minutes at 4°C. Anti-HA antibody was added to the precleaned samples and left for 2 hours on a rotating wheel at 4°C. To precipitate the antigen-antibody complexes, 50% bead suspension was added to the samples and incubated for 1 hour at 4°C. The pellets were washed with lysis buffer, and the samples were boiled in 2× SDS-PAGE and used for immunoblot analysis. For the oligomerization assay, U2OS UNC45A-KO cells were transiently transfected with either HA-tagged wild-type or mutant UNC45A constructs alone or cotransfected with His- and HA-tagged wild-type and mutant UNC45A constructs. The cells were lysed in EDTA-free EB4 lysis buffer and centrifuged for 40 minutes at 16,000*g*. Supernatants were supplemented with 17.5 mM imidazole (MilliporeSigma, 1202) and precleaned with Protein A Sepharose by rotating for 30 minutes at 4°C. Samples were mixed with 50% Ni-NTA Agarose beads (Invitrogen, R901-01), and binding of His-tagged proteins to Ni-NTA Agarose was performed for 1.5 hours at 4°C on a rotating wheel. The beads were pelleted and washed with EDTA-free EB4 lysis buffer supplemented with 25 mM imidazole and subsequently 400 mM NaCl. Input and pull-down samples were boiled in SDS-PAGE sample buffer and used for immunoblot analysis. The intensity of bands was analyzed using ImageJ, and the values were normalized to the His-tag protein levels.

### IF microscopy and image analysis.

For IF labeling, cells were fixed with either 4% paraformaldehyde solution (PFA) for 30 minutes at room temperature or with 100% methanol for 5 minutes at –20°C. To inhibit the proteasome, cells were treated with 10 μM MG-132 (MilliporeSigma, M7449) for 4 hours at 37°C. PFA-fixed cells were permeabilized with 0.05% Saponin (FLUKA, 84510) in blocking buffer (Cytoskeleton buffer, CB buffer: 20 mM Pipes, pH 6.8, 300 mM NaCl, 10 mM EGTA, 10 mM glucose, and 10 mM MgCl_2_, supplemented with 0.5 M NH_4_Cl and 10% goat serum) and incubated with primary antibodies for 1 hour at room temperature. The coverslips were washed with washing buffer (CB buffer, 0.5 M NH_4_Cl), incubated with secondary antibody for 1 hour at room temperature, stained with HOECHST, and mounted in Mowiol. The cells were analyzed by the Axio Imager M1 epifluorescence microscope (Carl Zeiss) with a 63× oil immersion objective (NA 1.4) equipped with a charge-coupled device camera (SPOT Xplore, Visitron Systems) and recorded with the VisiView 2.03 software (Visitron Systems). Imaging of single confocal planes of U2OS cells was performed with a confocal fluorescence microscope (LSM980, Carl Zeiss) from the Biooptics Core Facility at the Medical University of Innsbruck, using a 63× glycerol objective (NA 1.3). Acquisition and deconvolution were done with the ZEN3.3 software (Carl Zeiss) and Huygens Professional Deconvolution and Analysis program (Scientific Volume Imaging). The images were adjusted for brightness and contrast using ImageJ. Colocalization calculations (Pearson’s R without threshold) were performed in Coloc 2 plugin for ImageJ, as described (72).

### Electron microscopy of CaCo2 models and patient biopsy material.

Specimen processing was performed as described. Briefly, for morphological analysis of ultrathin resin sections by transmission electron microscopy (EM) biopsies were fixed with glutaraldehyde (2.5%), followed by osmium tetroxide (0.5%) (5). CaCo2 cells cultured for 21–28 days on 24 mm filters (Costar Transwell; pore size of 0.4 μm; Corning) were high-pressure frozen and freeze substituted (73). Immunogold labeling was performed on thawed ultrathin cryosections obtained from 14-day-old, fully polarized cell cultures chemically fixed with 4% formaldehyde (73). Antibodies used included rabbit anti-NHE3 (1:150; HPA036669 from MilliporeSigma), rabbit anti-Rab11a (1:100; from Invitrogen, 715300), and goat anti-DPPIV/CD26 (1:10; AF1180 from R&D Systems, Bio-Techne), along with appropriate secondary antibodies (British Biocell International, EM.GAR5/1, EM.GAR10/1, and EM.RAG 15/2) conjugated to 5, 10, or 15 nm colloidal gold, respectively (British Biocell International). Standard processing for scanning EM of chemically fixed, filter-grown CaCo2 monolayers (21–28 days) included critical point drying and sputter coating (73). Digital EM images were optionally adjusted with Photoshop CS6 (Adobe) to improve image contrast, brightness, grayscale, and sharpness.

### Tfn uptake and recycling.

U2OS, UNC45A-KO, and stably complemented cells were grown for 48 hours on poly-d-lysine–coated (Thermo Fisher Scientific, A3890401) glass coverslips and subsequently transferred to serum-free medium. To monitor early endocytic events, cells were kept on ice for 20 minutes followed by incubation with 5 μg/mL Alexa Fluor 488 ChromPure Human Transferrin (Tfn–Alexa Fluor 488; Jackson ImmunoResearch, 009-540-050) in cold FBS-free medium for 30 minutes. The cells were then washed and either fixed with 4% PFA (time 0) or transferred to FBS-supplemented medium, shifted to 37°C, and fixed after 5, 10, or 20 minutes. Tfn recycling was studied by exposing starved cells to Tfn–Alexa Fluor 488 for 1 hour at 37°C. After saturation, unbound Tfn–Alexa Fluor 488 was washed out, and cells representing time point 0 were fixed with 4% PFA; others were incubated for another 40 minutes or 3 hours in complete medium prior to fixation. To evaluate possible involvement of catabolic processes, 200 μg/mL leupeptin was added to cells 1 hour before Tfn incubation. Pharmacological NMII inhibition was done by adding 50 μM para-aminoblebbistatin [(-)Blebb; Motorpharma Ltd., DR-Am-89] after saturating cells with Tfn–Alexa Fluor 488.

### Statistics.

The results are presented as mean ± SD. Statistical comparisons were calculated using 2-tailed Student’s *t* test. *P* values are denoted as significant if less than 0.05.

### Study approval.

Written informed consent for molecular genetic studies and publication of data was obtained, and the ethics committee of the Medical University of Innsbruck approved the study (No. AN2016-002, 10/2021).

### Data availability.

Original images of immunoblots are provided in the unedited gel images PDF file, and raw data values are supplied in the Supporting Data Values XLS file.

## Author contributions

TM, MWH, ARJ, LAH, and TV conceived and designed the study. AMD, RA, MP, and NCB provided clinical data and managed cases. ARJ performed genetic analysis. SW, KK, KP, MWH, and TV conducted experiments and acquired data. Quantitative analysis, including data analysis and statistical evaluation, was contributed by ARP and VD. SW, KK, JV, KP, GFV, HHU, FMR, TM, MWH, ARJ, LAH, and TV analyzed and interpreted data. SW and TV assembled figures. SW, ARJ, MWH, LAH, and TV wrote the manuscript. All authors reviewed and agreed on the manuscript.

## Supplementary Material

Supplemental data

Unedited blot and gel images

Supporting data values

## Figures and Tables

**Table 1:**
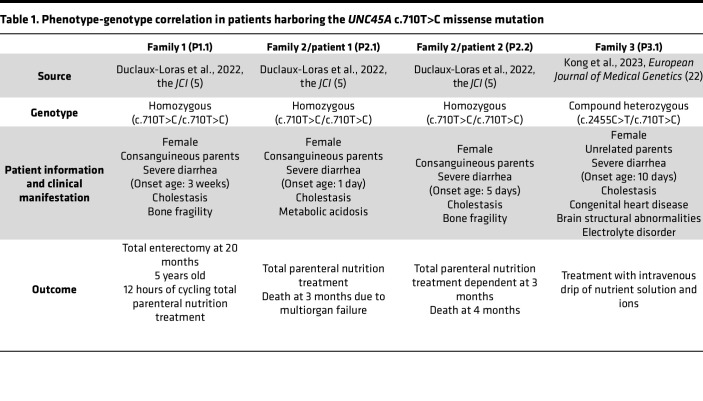
Phenotype-genotype correlation in patients harboring the *UNC45A* c.710T>C missense mutation

## Version 1 03/24/2025

Electronic publication
