# Supplementary material for: Altered chaperone–nonmuscle myosin II interactions drive pathogenicity of the UNC45A c.710T>C variant in osteo-oto-hepato-enteric syndrome
Source: JCI Insight. 2025 Mar 24;10(6):e185508. doi: 10.1172/jci.insight.185508 (PMC11949031; doi:10.1172/jci.insight.185508)

Altered Chaperone-Non-Muscle Myosin II Interactions Drive Pathogenicity of the *UNC45A* c.710T>C Variant in Osteo-Oto-Hepato-Enteric Syndrome

Stephanie Waich,[1] Karin Kreidl,[1,2] Julia Vodopiutz,[3,4] Arzu Meltem Demir,[5,6] Adam R. Pollio,[1] Vojtěch Dostál,[1] Kristian Pfaller,[7] Marianna Parlato,[8] Nadine Cerf-Bensussan,[8] Rüdiger Adam,[9] Georg F. Vogel,[1,2] Holm H. Uhlig,[10] Frank M. Ruemmele,[11] Thomas Müller,[2] Michael W. Hess,[7] Andreas R. Janecke,[2,12] Lukas A. Huber,[1] and Taras Valovka[1,2]

[1]Institute of Cell Biology, Biocenter, Medical University of Innsbruck, 6020 Innsbruck, Austria.

[2]Department of Paediatrics I, Medical University of Innsbruck, 6020 Innsbruck, Austria.

[3]Department of Paediatrics and Adolescent Medicine, Division of Paediatric Pulmonology, Allergology and Endocrinology, Comprehensive Center for Paediatrics, Medical University of Vienna, 1090 Vienna, Austria.

[4]Full-Member of the ERN – BOND and Vienna Bone and Growth Center, 1090 Vienna, Austria.

[5]Ankara Child Health and Diseases, Training and Research Hospital, Department of Paediatric Gastroenterology, Ankara, Türkiye.

[6]Ankara University School of Medicine, Department of Paediatrics, Division of Paediatric Gastroenterology, Hepatology and Nutrition, Ankara, Türkiye.

[7]Institute of Histology und Embryology, Medical University of Innsbruck, 6020 Innsbruck, Austria.

[8]Université Paris Cité, Imagine Institute, Laboratory of Intestinal Immunity, INSERM, UMR 1163, Paris, France.

[9]University Children's Hospital, Paediatric Gastroenterology, Hepatology and Nutrition, Medical Faculty Mannheim, Heidelberg University, Mannheim, Germany.

[10]Nuffield Department of Clinical Medicine, Experimental Medicine Division, and Department of Paediatrics, and Oxford Biomedical Research Centre, University of Oxford, Oxford, United Kingdom.

[11]Université Paris Cité, Faculté de Santé, UFR de Médicine, APHP, Hôpital Universitaire Necker Enfants Malades, Departement de Gastronenterology Pediatrique, Institut IMAGINE INSERM UMR 1163, Paris, France.

[12]Institute of Human Genetics, Medical University of Innsbruck, 6020 Innsbruck, Austria.

Supplemental Figure 1. UNC45A p.Leu237Pro shows decreased resistance to heat-induced unfolding.

A) Dot-blot analysis of unheated (37°C) and heated (45.5-70°C) UNC45A wild-type and mutant samples with anti-HA antibody.

B) Thermal shift assay aggregation curves. The quantification of dot blots was performed with densitometry analysis using ImageJ, and T_{agg} was calculated with the R programme as described in the Methods section. Values are mean ± SD of at least four independent experiments.

Supplemental Figure 2. Chaperone activity and actomyosin complex formation are restored by UNC45A p.Leu237Pro in U2OS UNC45A KO cells.

A) NMIIA-associated structures were visualized in U2OS parental, UNC45A KO and UNC45A KO cells transiently transfected with HA-tagged UNC45A wild-type and mutant constructs. Cells were labelled with antibodies directed against the HA-tag (green) and NMIIA (red), while nuclei were stained with HOECHST (blue). White arrowheads indicate the normal structures and distribution of NMIIA in U2OS parental and transfected cells, while yellow arrowheads indicate the dispersed NMIIA staining observed in UNC45A KO cells (upper panels). In addition, NMIIA-positive aggregates were prominently

visible in MG-132-treated UNC45A KO cells, as indicated by yellow arrowheads (lower panels). Scale bars, 20 µm.

B) Effects of UNC45A deficiency and the p.Leu237Pro UNC45A on actin cytoskeleton organization in U2OS cells. UNC45A KO cells were transiently transfected with HA-tagged UNC45A wild-type and mutant constructs, and labelled with an antibody directed against the HA-tag (green), phalloidin for the actin cytoskeleton (red) and HOECHST for nuclei (blue). Yellow arrowheads represent the atypical distribution and structures of actin in UNC45A deficient U2OS cells, compared to normal cytoskeleton organization (white arrowheads). Scale bars, 20 µm.

Supplemental Figure 3. The p.Leu237Pro UNC45A chaperone does not affect microtubule organization in U2OS cells.

Immunofluorescence images of U2OS parental, UNC45A KO and stably complemented UNC45A wild-type and mutant cells. The cells were stained with a specific antibody for alpha-tubulin (green), and HOECHST was used to label the nuclei (blue). Random cellular fields were 10x magnified and are presented in the lowest panel. Scale bars, 20 µm.

Supplemental Figure 4. Formation and maturation of focal adhesions is restored by UNC45A p.Leu237Pro in UNC45A KO cells.

U2OS parental, UNC45A KO and UNC45A KO cells transiently transfected with HA-tagged UNC45A wild-type and mutant constructs, were labelled with antibodies directed against the HA-tag (green), and the early and late focal adhesion proteins paxillin and zyxin, respectively (red). HOECHST was used as a marker for nuclei (blue). Yellow arrowheads indicate atypical focal adhesions in UNC45A deficient cells and are compared to normal ones observed in parental U2OS as well as in wild-type and mutant UNC45A expressing UNC45A KO cells (white arrowheads). Scale bars, 20 µm.

Supplemental Figure 5. Cell surface binding and early internalization of Tfn appear intact in UNC45A deficient and UNC45A mutant expressing cells

A) Scheme showing the experimental setup for Tfn internalization.

B) Epifluorescence microscopy of U2OS cells incubated and pulse-chased with Tfn-A488 (green) shows normal surface binding and internalization in all U2OS genotypes investigated. Nuclei were stained with HOECHST (blue). Scale bars, 20 µm.

Supplemental Figure 6. The Tfn trafficking and processing phenotype is not associated with defective lysosomal degradation.

A) Scheme of the Tfn recycling assay in cells treated with the lysosomal inhibitor leupeptin (Leu).

B) Representative images of U2OS cells subjected to Tfn recycling in the presence of 200 µg/ml leupeptin (Leu) do not differ from the phenotype shown in Figure 6. The inhibitor was present throughout the experiment from 1 hour prior to Tfn saturation. The internalized Tfn-Alexa488 is shown in green, and nuclei were labelled with HOECHST (blue). Yellow arrow heads mark abnormal retention of Tfn-Alexa488 after 40 min and 3 hours of recycling in UNC45A KO and UNC45A MUT cells. Scale bars, 10 µm.

C) Quantification of Tfn-A488 positive cells. Data were analyzed as described in Figure 6B. Significance: * $p < 0.05$, ** $p < 0.01$, *** $p < 0.001$.

Supplemental Figure 7. Confocal immunofluorescence microscopy of Tfn-harbouring endocytic compartments in U2OS cell lines after cargo uptake (0 minutes) and recycling (40 minutes).

Magnified regions of immunofluorescence images were taken from Figure 7 and are presented as black and white single channels for Tfn and the specified markers. Note the persistent Tfn accumulation in EEA1-, Rab11a-, and Lamp1-positive compartments after 40 minutes of recycling in UNC45A KO and p.Leu237Pro mutant cells (yellow arrowheads). Scale bars, 20 µm.

Supplemental Figure 8. Quantification of Tfn-Alexa488 co-localization with EEA1-, Rab11-, or Lamp1-positive compartments in U2OS, UNC45A KO and reconstituted cells presented in Figure 7.

The probability of co-localization between Tfn-Alexa488 signal and the signal of different cellular compartments (EEA1, Rab11, and Lamp1) was estimated using the Pearson's R (without threshold) correlation coefficient. Each dot on the graphs corresponds to a single cell and the graphs were established from three independent experiments.

Supplemental Figure 9. Tfn recycling patterns in U2OS cells concurrently treated with inhibitors for NMII and lysosomal degradation.

A) Scheme of Tfn recycling upon treatment with leupeptin (Leu) and para-aminoblebbistatin (Blebb).

B) No major differences are seen in representative images of U2OS cells that were subjected to Tfn uptake and recycling in the presence of both, 200 µg/ml Leu and 50 µM Blebb. The internalized Tfn-Alexa488 is shown in green, and nuclei were visualized with HOECHST (blue). Scale bars, 10 µm.

C) Quantification of Tfn-Alexa488 positive cells. Data were analyzed applying two-tailed Student's t-Test.* $p < 0.05$, ** $p < 0.01$, *** $p < 0.001$. For a more detailed description, see Figure 6C.

Supplemental Figure 10. Genetically engineered CaCo2 cell models recapitulate in vitro the subcellular phenotype of MVID-like enteropathy seen in patient P1.1 carrying UNC45A p.Leu237Pro variant.

A) Western blot showing protein levels of UNC45A in CaCo2 parental WT, UNC45A KO and stably complemented UNC45A wild-type and mutant cells. Beta-actin was used as a loading control.

B) Electron micrographs of enterocytes from duodenal biopsies versus >21 day old, fully polarized and differentiated CaCo2 monolayer cultures (UNC45A KO, p.Leu237Pro). Ultrastructural alterations comprise (i) brush border microvilli (double arrow-heads), (ii) partially enlarged (autophago)lysosomes

(cross: +), (iii) the occurrence of microvillous inclusions (arrows)and basolateral microvilli assemblies (double arrows), as well as (iv) abnormal subapical tubulo-vesicles (arrow-heads); g=glycogen. Scale bars: 2.5 µm (left lane), 500 nm (middle/right lanes). Note that scale bars in low-mag images of CaCo2 cells mark also the plane of the culture substrate.

Supplemental Figure 11. Subcellular phenotype rescued by re-expression of UNC45A WT protein in CaCo2 UNC45A KO cells plus illustration of cell surface morphology and brush border DPPIV-immunogold labelling.

A) Transmission electron microscopy shows normal ultrastructure with (i) regular brush border microvilli (double arrow-heads), (ii) lysosomes of normal size and shape (cross: +), complete absence of both (iii) ectopic, intra- or paracellular microvilli, and of (iv) subapical tubulo-vesicles in UNC45A wild-type expressing cells resembling CaCo2 parental WT cells; g=glycogen, n=nuclues. Scale bars: 2.5 µm (left lane), 500 nm (middle/right lanes).

B) Scanning electron microscopy of scarce and/or partially irregularly shaped apical microvilli in p.Leu237Pro variant expressing and UNC45A KO cells versus normal, dense brush border in UNC45A wild-type expressing and control cells. Scale bars: 2 µm.

C) Comparable DPPIV- immunogold label (arrow-heads) associated with the apical microvilli of all genotypes. Scale bars: 500 nm.

Supplemental Figure 12. Rab11- and NHE3-immunogold labelling of polarized, genetically engineered and control CaCo2.

A) Numerous Rab11-immunogold particles (arrow-heads) in p.Leu237Pro variant expressing and UNC45A KO cells, frequently associated with abnormal subapical tubulo-vesicles, versus scarce, though distinct Rab11-immunogold label in UNC45A wild-type expressing and CaCo2 control cells. Scale bars: 200 nm.

B) NHE3-immunogold particles (arrow-heads) associated with subapical vesicles in p.Leu237Pro variant expressing and UNC45A KO cells, versus normal localization predominantly on the brush border in UNC45A wild-type expressing and CaCo2 control cells. Scale bars: 500 nm.

Supplemental Figure 1. UNC45A p.Leu237Pro shows decreased resistance to heat-induced unfolding.

A. Dot-blot analysis of unheated (37°C) and heated (45.5-70°C) UNC45A wild-type and mutant samples with anti-HA antibody.
B. Thermal shift assay aggregation curves. The quantification of dot blots was performed with densitometry analysis using ImageJ, and T_{agg} was calculated with the R programme as described in the Methods section. Values are mean ± SD of at least four independent experiments.

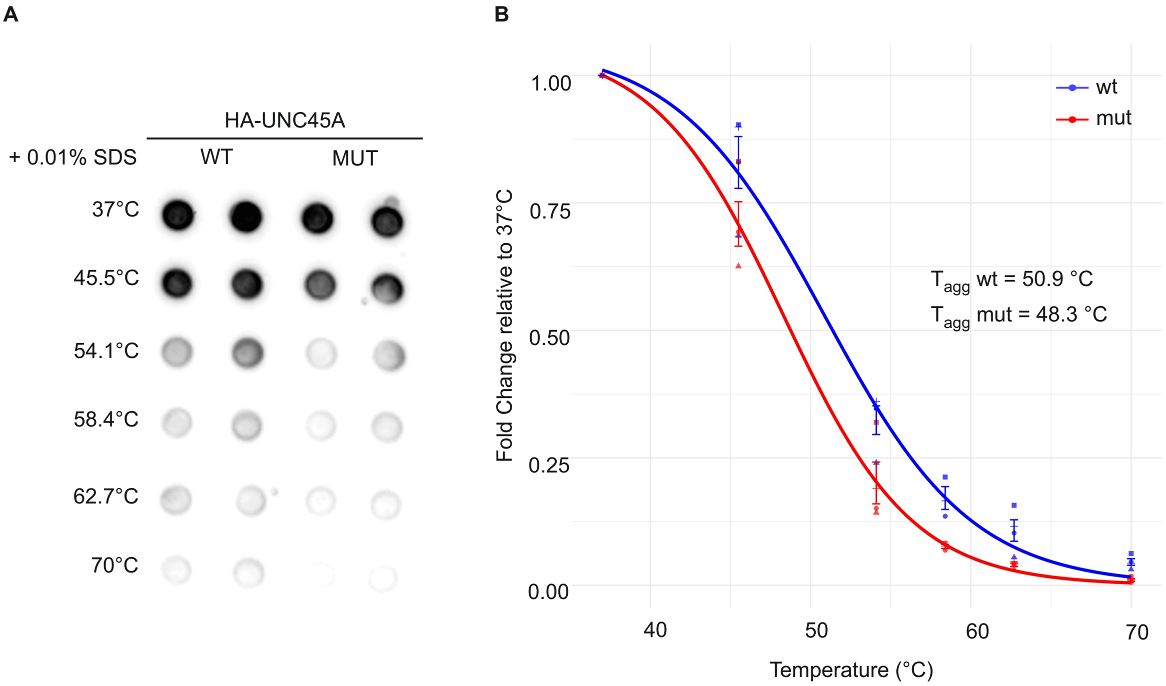

Supplemental Figure 2. Chaperone activity and actomyosin complex formation are restored by UNC45A p.Leu237Pro in U2OS UNC45A KO cells.

A. NMIIA-associated structures were visualized in U2OS parental, UNC45A KO and UNC45A KO cells transiently transfected with HA-tagged UNC45A wild-type and mutant constructs. Cells were labelled with antibodies directed against the HA-tag (green) and NMIIA (red), while nuclei were stained with HOECHST (blue). White arrowheads indicate the normal structures and distribution of NMIIA in U2OS parental and transfected cells, while yellow arrowheads indicate the dispersed NMIIA staining observed in UNC45A KO cells (upper panels). In addition, NMIIA-positive aggregates were prominently visible in MG-132-treated UNC45A KO cells, as indicated by yellow arrowheads (lower panels). Scale bars, 20 µm.
B. Effects of UNC45A deficiency and the p.Leu237Pro UNC45A on actin cytoskeleton organization in U2OS cells. UNC45A KO cells were transiently transfected with HA-tagged UNC45A wild-type and mutant constructs, and labelled with an antibody directed against the HA-tag (green), phalloidin for the actin cytoskeleton (red) and HOECHST for nuclei (blue). Yellow arrowheads represent the atypical distribution and structures of actin in UNC45A deficient U2OS cells, compared to normal cytoskeleton organization (white arrowheads). Scale bars, 20 µm.

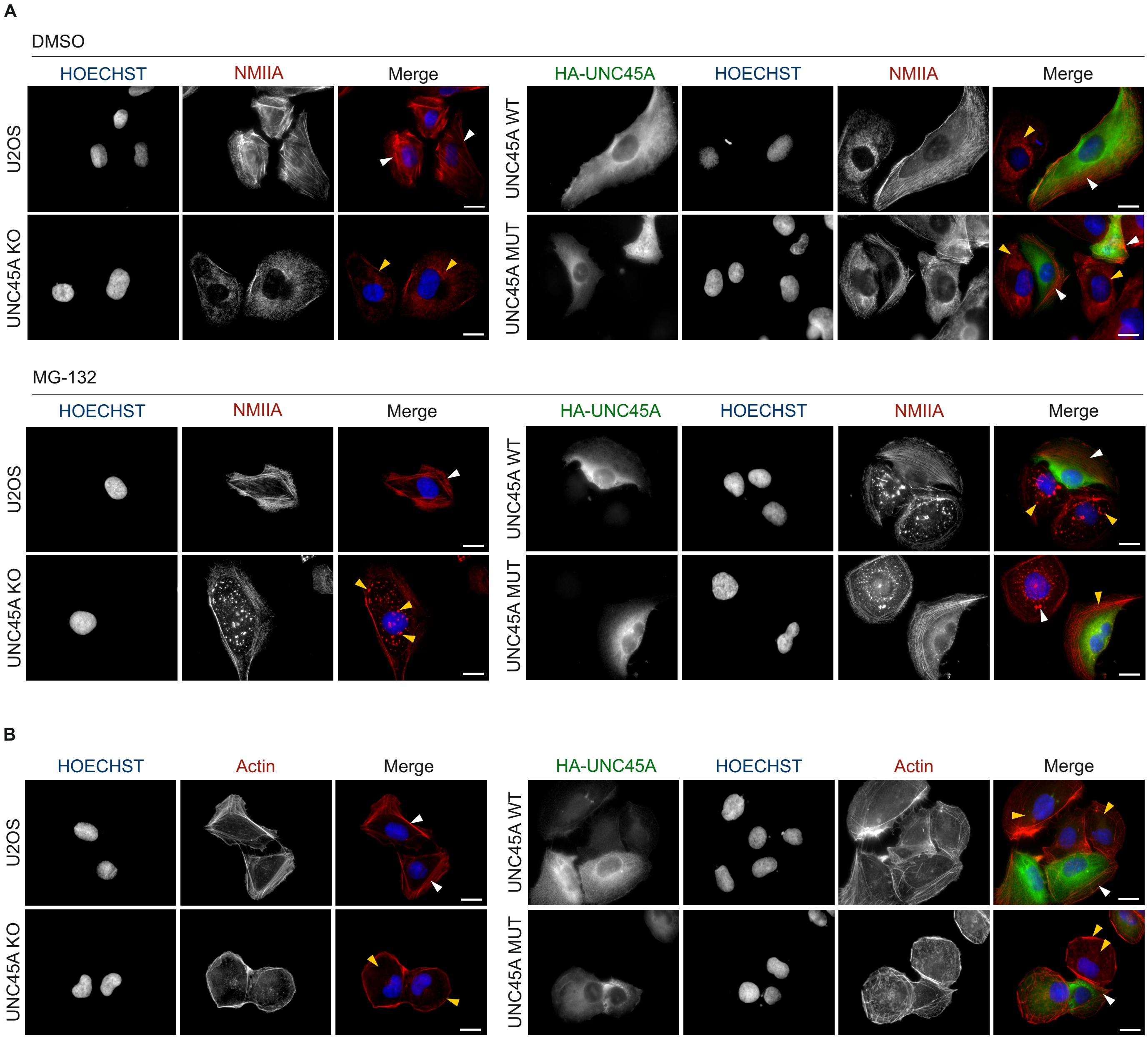

Supplemental Figure 3. The p.Leu237Pro UNC45A chaperone does not affect microtubule organization in U2OS cells.

Immunofluorescence images of U2OS parental, UNC45A KO and stably complemented UNC45A wild-type and mutant cells. The cells were stained with a specific antibody for alpha-tubulin (green), and HOECHST was used to label the nuclei (blue). Random cellular fields were 10x magnified and are presented in the lowest panel. Scale bars, 20 μm.

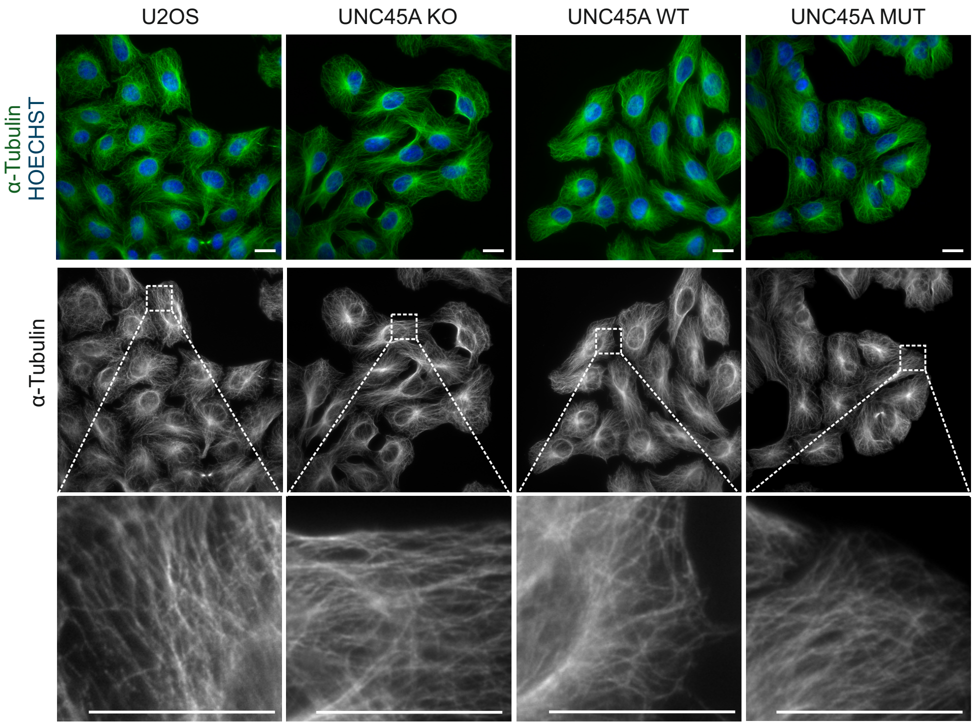

Supplemental Figure 4. Formation and maturation of focal adhesions is restored by UNC45A p.Leu237Pro in UNC45A KO cells.

U2OS parental, UNC45A KO and UNC45A KO cells transiently transfected with HA-tagged UNC45A wild-type and mutant constructs, were labelled with antibodies directed against the HA-tag (green), and the early and late focal adhesion proteins paxillin and zyxin, respectively (red). HOECHST was used as a marker for nuclei (blue). Yellow arrowheads indicate atypical focal adhesions in UNC45A deficient cells and are compared to normal ones observed in parental U2OS as well as in wild-type and mutant UNC45A expressing UNC45A KO cells (white arrowheads). Scale bars, 20 µm.

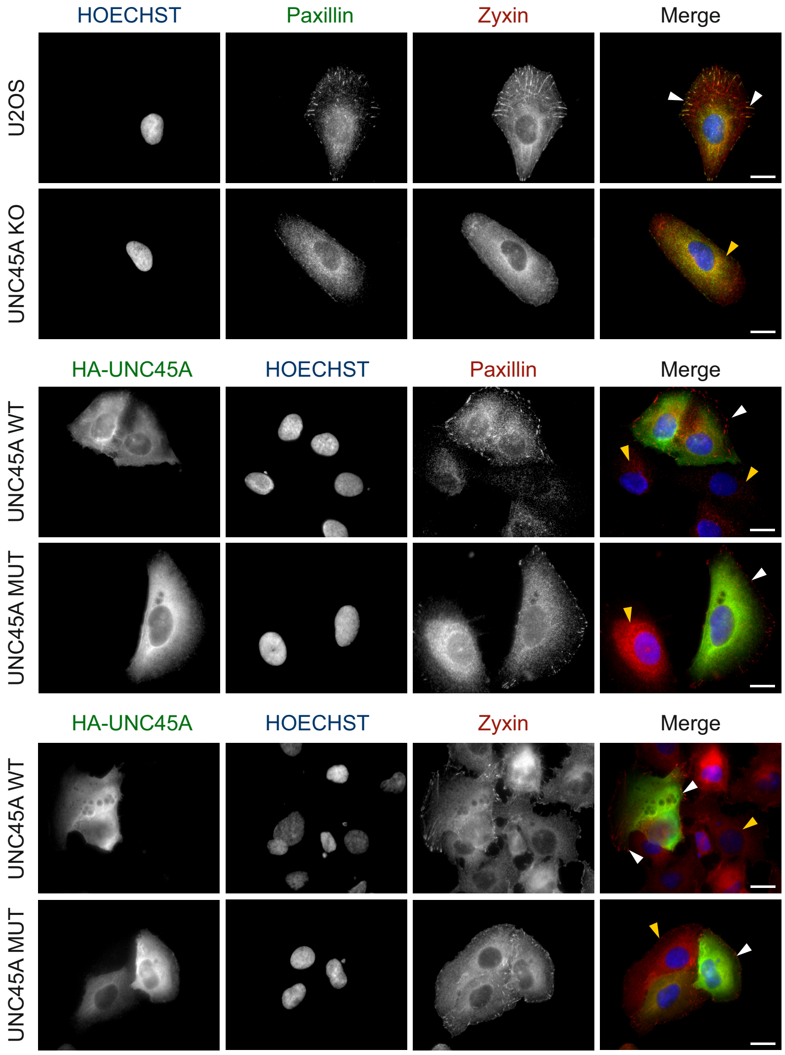

Supplemental Figure 5. Cell surface binding and early internalization of Tfn appear intact in UNC45A deficient and UNC45A mutant expressing cells

A. Scheme showing the experimental setup for Tfn internalization.
B. Epifluorescence microscopy of U2OS cells incubated and pulse-chased with Tfn-Alexa488 (green) shows normal surface binding and internalization in all U2OS genotypes investigated. Nuclei were stained with HOECHST (blue). Scale bars, 20 µm.

A

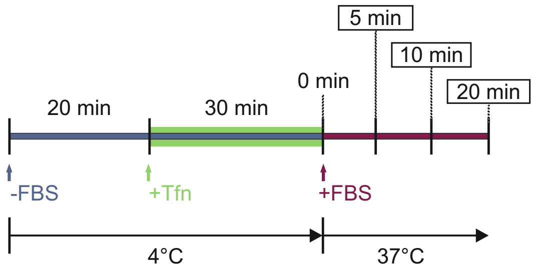

B

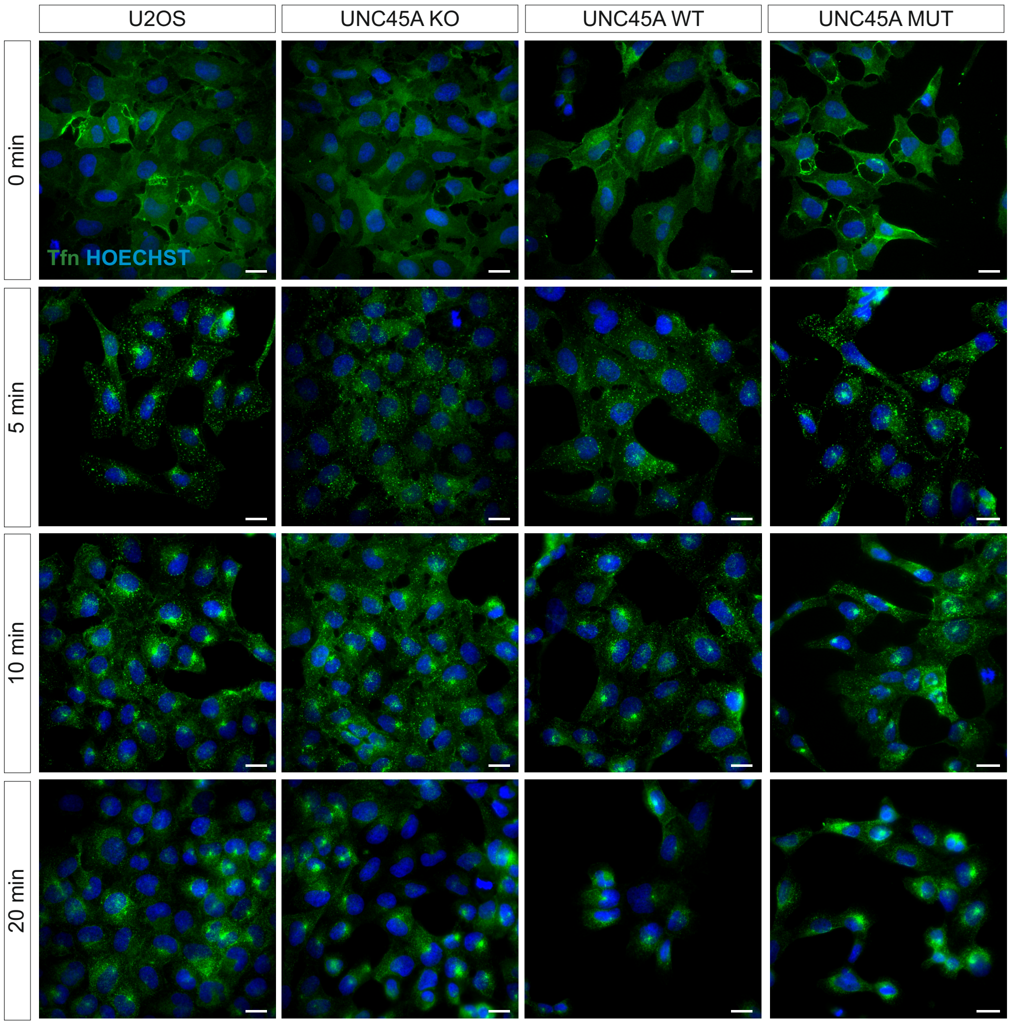

Supplemental Figure 6. The Tfn trafficking and processing phenotype is not associated with defective lysosomal degradation.

A. Scheme of the Tfn recycling assay in cells treated with the lysosomal inhibitor leupeptin (Leu).
B. Representative images of U2OS cells subjected to Tfn recycling in the presence of 200 µg/ml leupeptin (Leu) do not differ from the phenotype shown in Figure 6. The inhibitor was present throughout the experiment from 1 hour prior to Tfn saturation. The internalized Tfn-Alexa488 is shown in green, and nuclei were labelled with HOECHST (blue). Yellow arrow heads mark abnormal retention of Tfn-Alexa488 after 40 min and 3 hours of recycling in UNC45A KO and UNC45A MUT cells. Scale bars, 10 µm.
C. Quantification of Tfn-Alexa488 positive cells. Data were analyzed as described in Figure 6B. Significance: * $p < 0.05$, ** $p < 0.01$, *** $p < 0.001$.

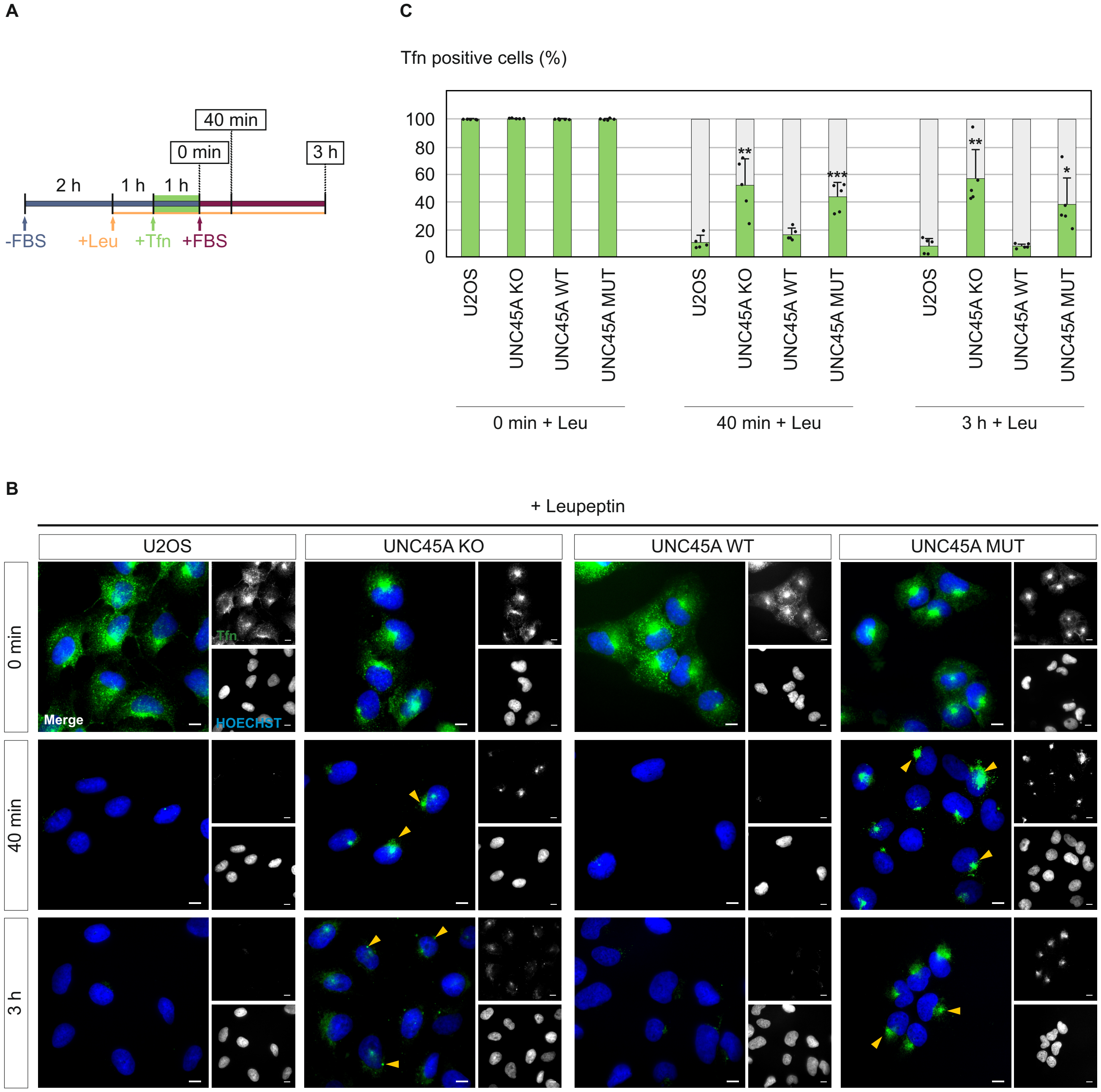

Supplemental Figure 7. Confocal immunofluorescence microscopy of Tfn-harbouring endocytic compartments in U2OS cell lines after cargo uptake (0 minutes) and recycling (40 minutes).

Magnified regions of immunofluorescence images were taken from Figure 7 and are presented as black and white single channels for Tfn and the specified markers. Note the persistent Tfn accumulation in EEA1-, Rab11a-, and Lamp1-positive compartments after 40 minutes of recycling in UNC45A KO and p.Leu237Pro mutant cells (yellow arrowheads). Scale bars, 20 µm.

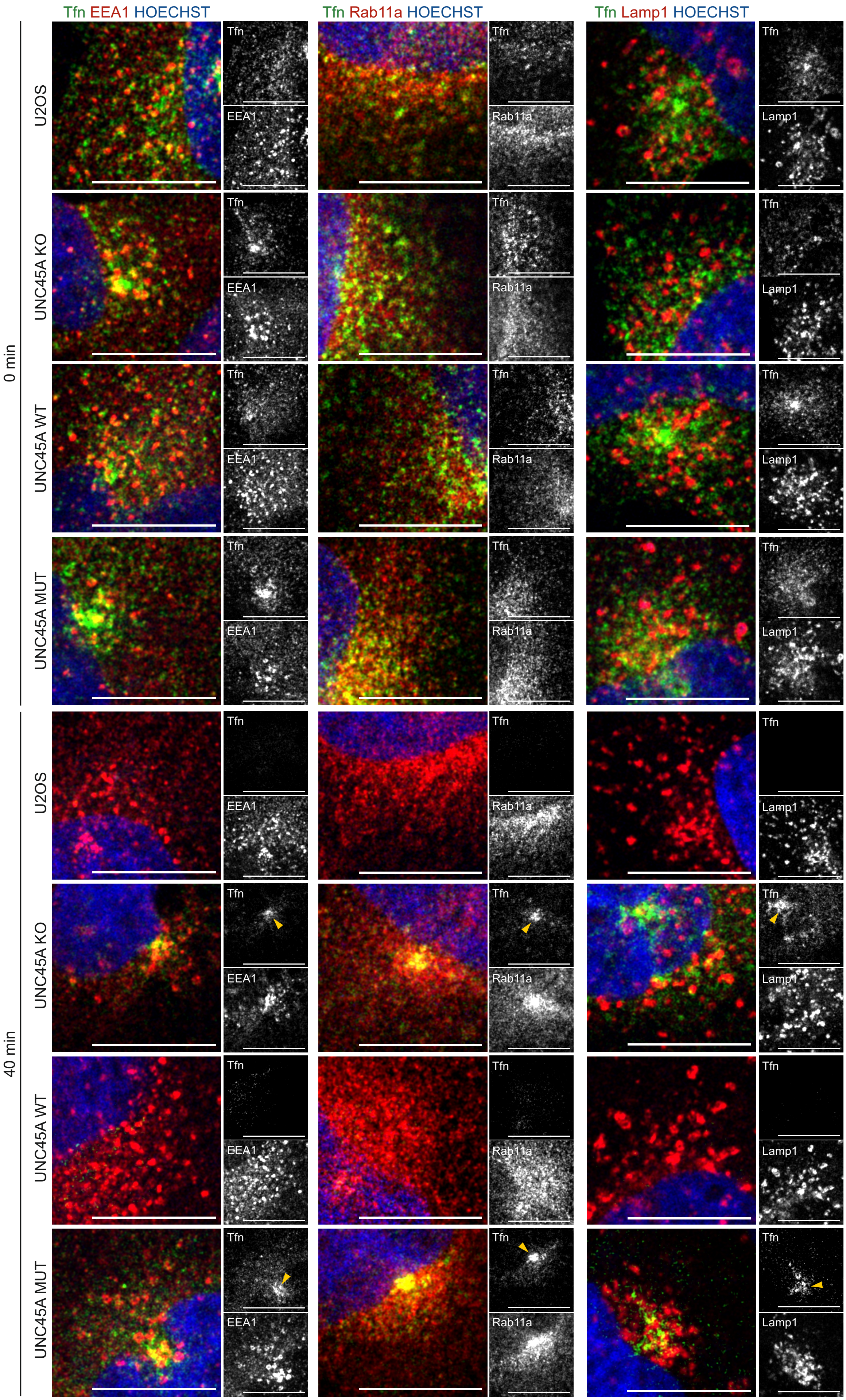

Supplemental Figure 8. Quantification of Tfn-Alexa488 co-localization with EEA1-, Rab11-, or Lamp1-positive compartments in U2OS, UNC45A KO and reconstituted cells presented in Figure 7.

The probability of co-localization between Tfn-Alexa488 signal and the signal of different cellular compartments (EEA1, Rab11, and Lamp1) was estimated using the Pearson's R (without threshold) correlation coefficient. Each dot on the graphs corresponds to a single cell and the graphs were established from three independent experiments.

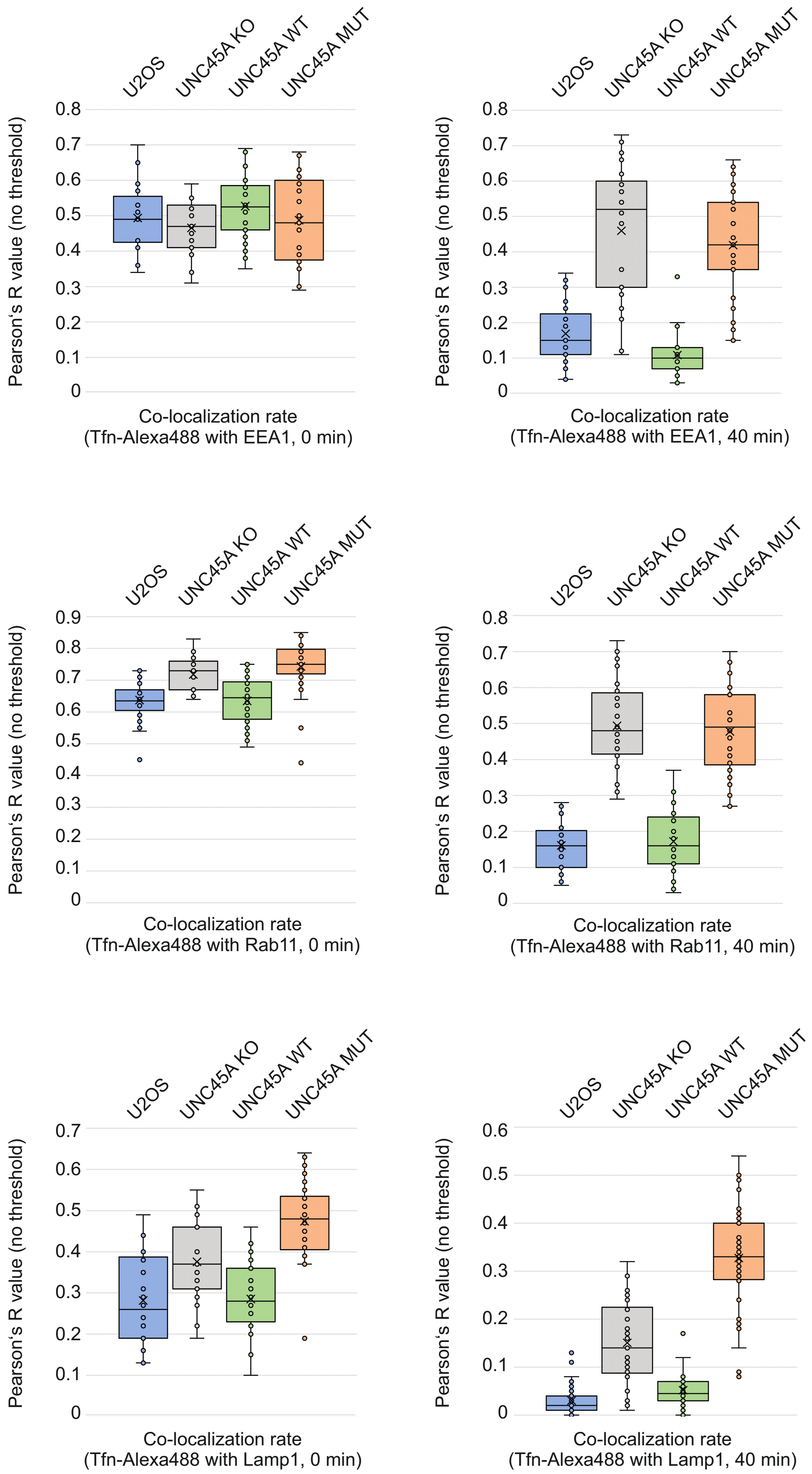

Supplemental Figure 9. Tfn recycling patterns in U2OS cells concurrently treated with inhibitors for NMII and lysosomal degradation.

A. Scheme of Tfn recycling upon treatment with leupeptin (Leu) and para-aminoblebbistatin (Blebb).
B. No major differences are seen in representative images of U2OS cells that were subjected to Tfn uptake and recycling in the presence of both, 200 µg/ml Leu and 50 µM Blebb. The internalized Tfn-Alexa488 is shown in green, and nuclei were visualized with HOECHST (blue). Scale bars, 10 µm.
C. Quantification of Tfn-Alexa488 positive cells. Data were analyzed applying two-tailed Student's t-Test.* $p < 0.05$, ** $p < 0.01$, *** $p < 0.001$. For a more detailed description, see Figure 6C.

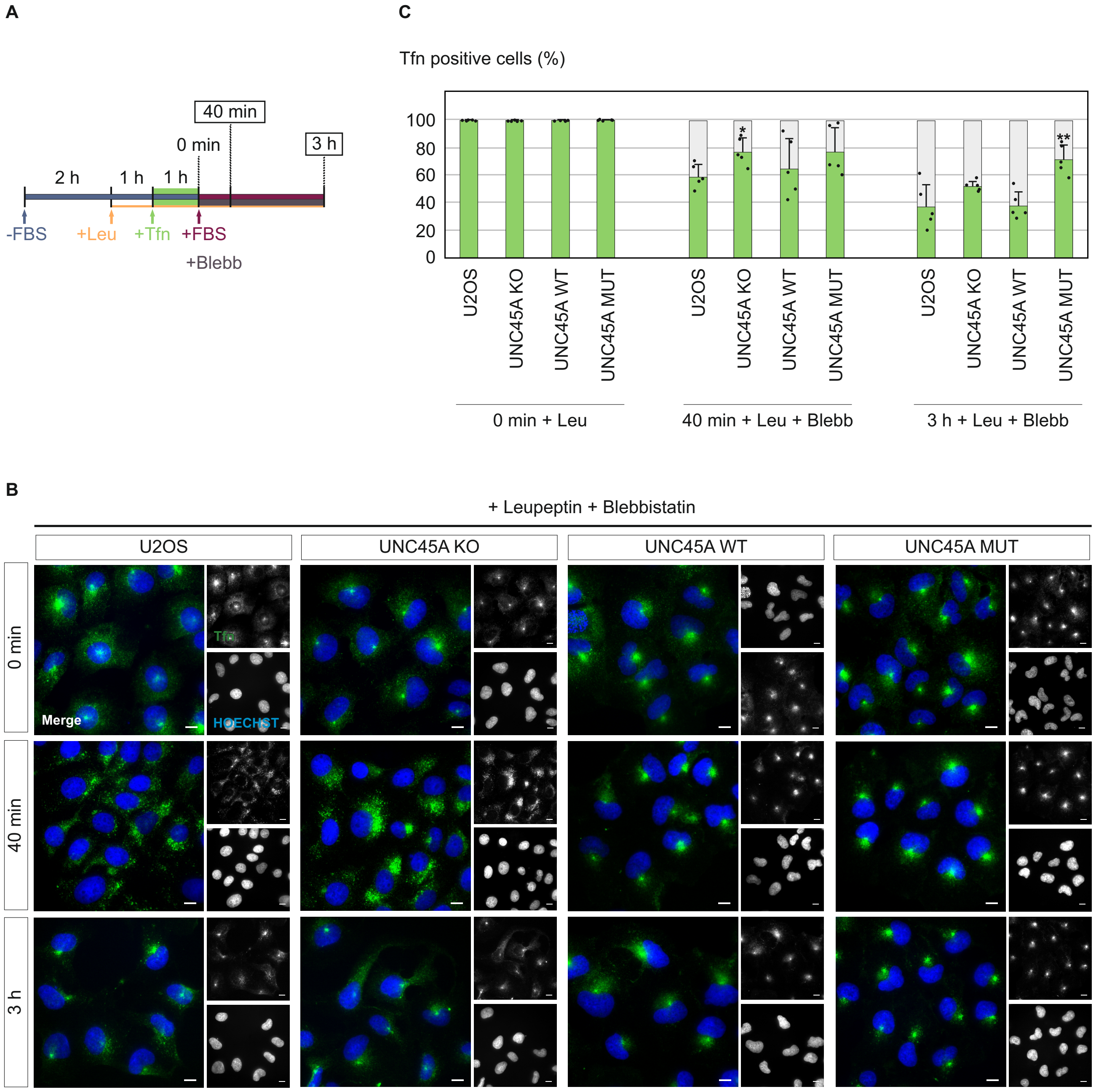

Supplemental Figure 10. Genetically engineered CaCo2 cell models recapitulate in vitro the subcellular phenotype of MVID-like enteropathy seen in patient P1.1 carrying UNC45A p.Leu237Pro variant.

A. Western blot showing protein levels of UNC45A in CaCo2 parental WT, UNC45A KO and stably complemented UNC45A wild-type and mutant cells. Beta-actin was used as a loading control.
B. Electron micrographs of enterocytes from duodenal biopsies versus >21 day old, fully polarized and differentiated CaCo2 monolayer cultures (UNC45A KO, p.Leu237Pro). Ultrastructural alterations comprise (i) brush border microvilli (double arrow-heads), (ii) partially enlarged (autophago)lysosomes (cross: +), (iii) the occurrence of microvillous inclusions (arrows)and basolateral microvilli assemblies (double arrows), as well as (iv) abnormal subapical tubulo-vesicles (arrow-heads); g=glycogen.
Scale bars: 2.5µm (left lane), 500nm (middle/right lanes). Note that scale bars in low-mag images of CaCo2 cells mark also the plane of the culture substrate.

A

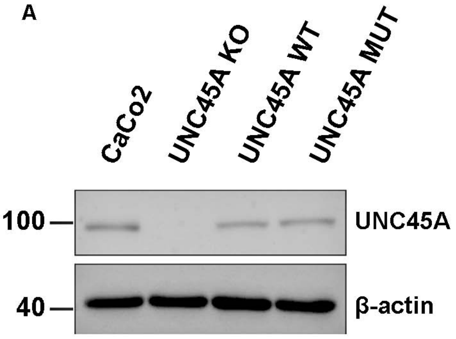

B

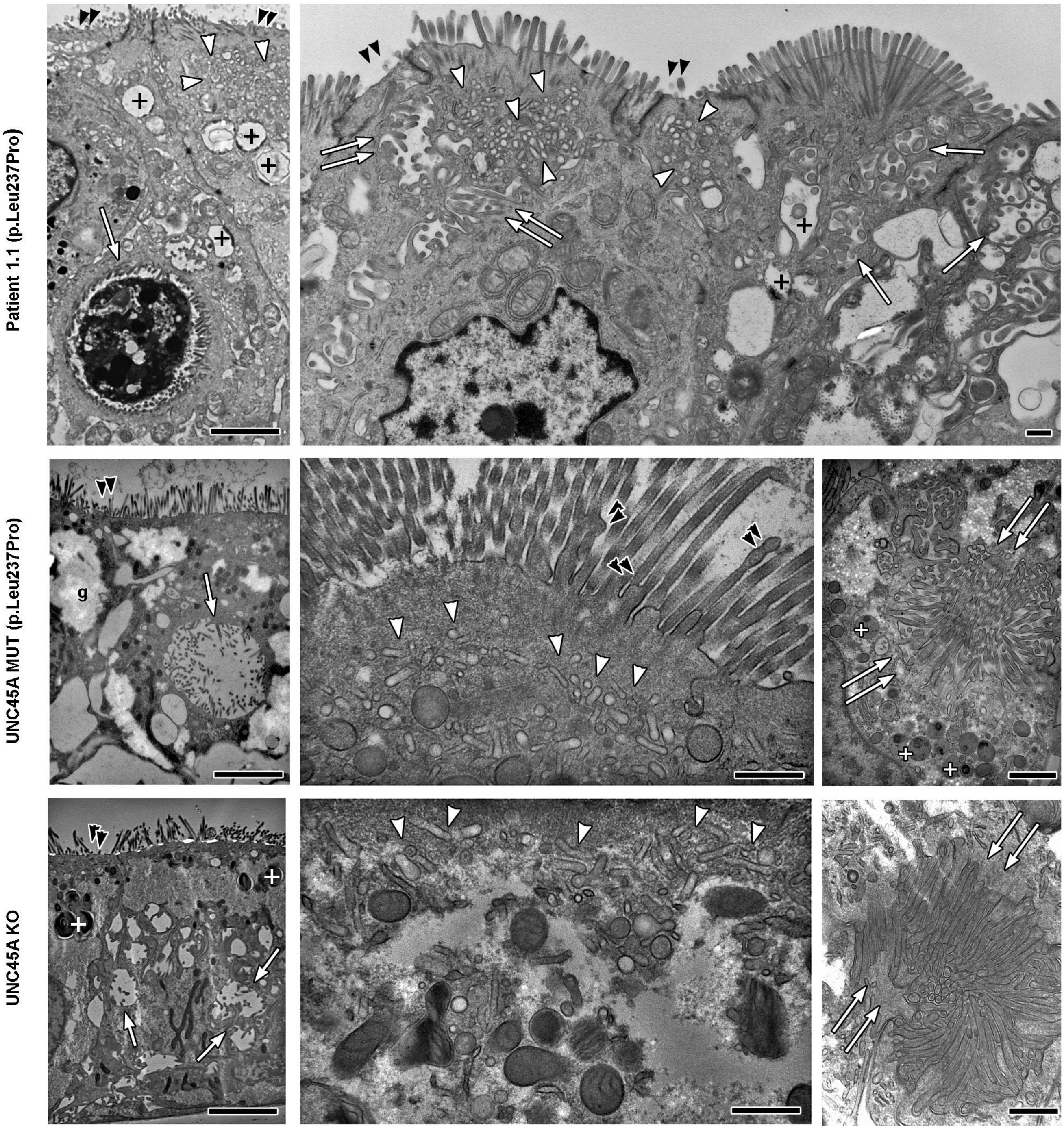

Supplemental Figure 11. Subcellular phenotype rescued by re-expression of UNC45A WT protein in CaCo2 UNC45A KO cells plus illustration of cell surface morphology and brush border DPPIV-immunogold labelling.

A. Transmission electron microscopy shows normal ultrastructure with (i) regular brush border microvilli (double arrow-heads), (ii) lysosomes of normal size and shape (cross: +), complete absence of both (iii) ectopic, intra- or paracellular microvilli, and of (iv) subapical tubulo-vesicles in UNC45A wild-type expressing cells resembling CaCo2 parental WT cells; g=glycogen, n=nuclues. Scale bars: 2.5µm (left lane), 500nm (middle/right lanes).

B. Scanning electron microscopy of scarce and/or partially irregularly shaped apical microvilli in p.Leu237Pro variant expressing and UNC45A KO cells versus normal, dense brush border in UNC45A wild-type expressing and control cells. Scale bars: 2µm.

C. Comparable DPPIV- immunogold label (arrow-heads) associated with the apical microvilli of all genotypes. Scale bars: 500nm.

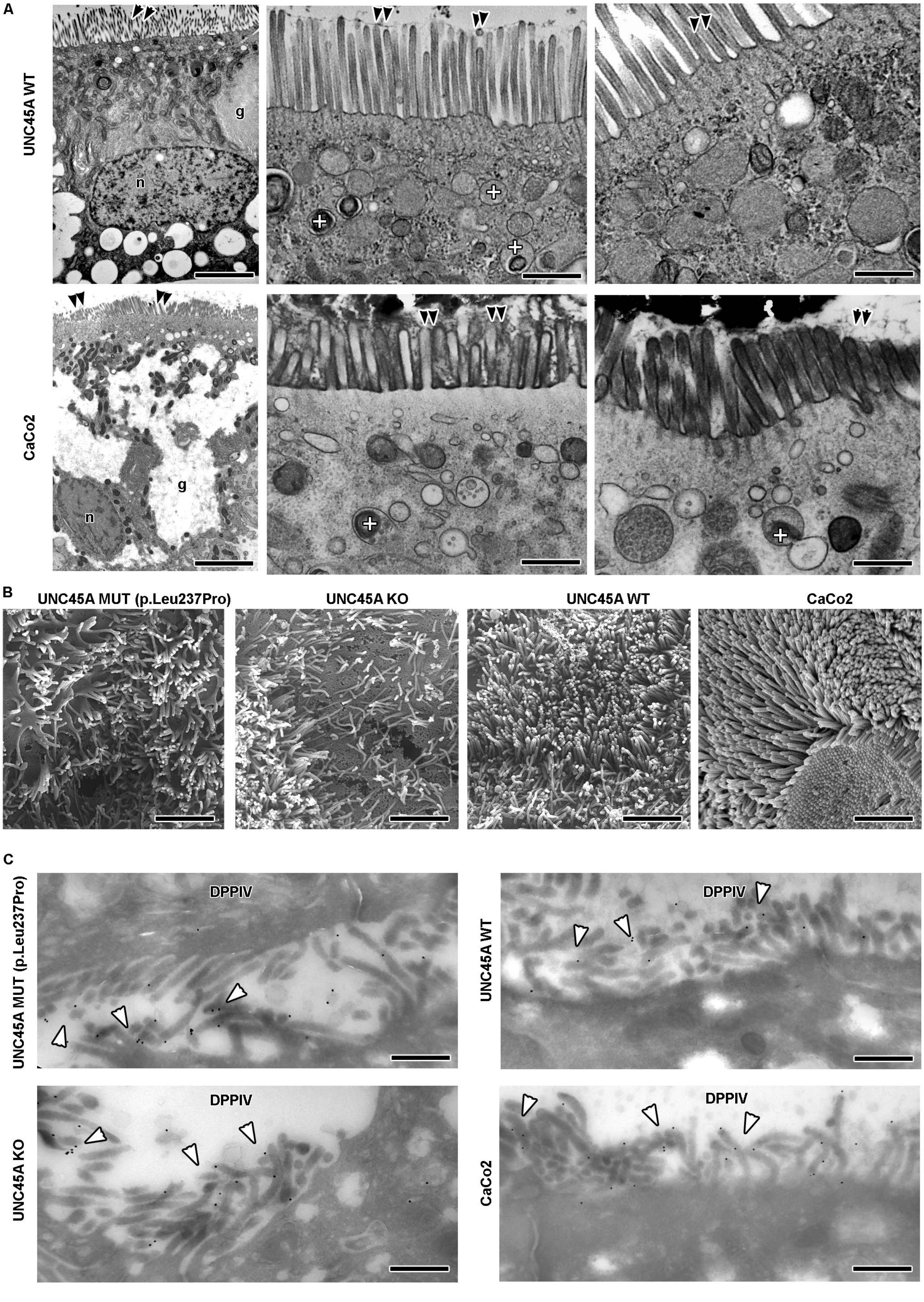

Supplemental Figure 12. Rab11- and NHE3-immunogold labelling of polarized, genetically engineered and control CaCo2.

A. Numerous Rab11-immunogold particles (arrow-heads) in p.Leu237Pro variant expressing and UNC45A KO cells, frequently associated with abnormal subapical tubulo-vesicles, versus scarce, though distinct Rab11-immunogold label in UNC45A wild-type expressing and CaCo2 control cells. Scale bars: 200nm.
B. NHE3-immunogold particles (arrow-heads) associated with subapical vesicles in p.Leu237Pro variant expressing and UNC45A KO cells, versus normal localization predominantly on the brush border in UNC45A wild-type expressing and CaCo2 control cells. Scale bars: 500nm.

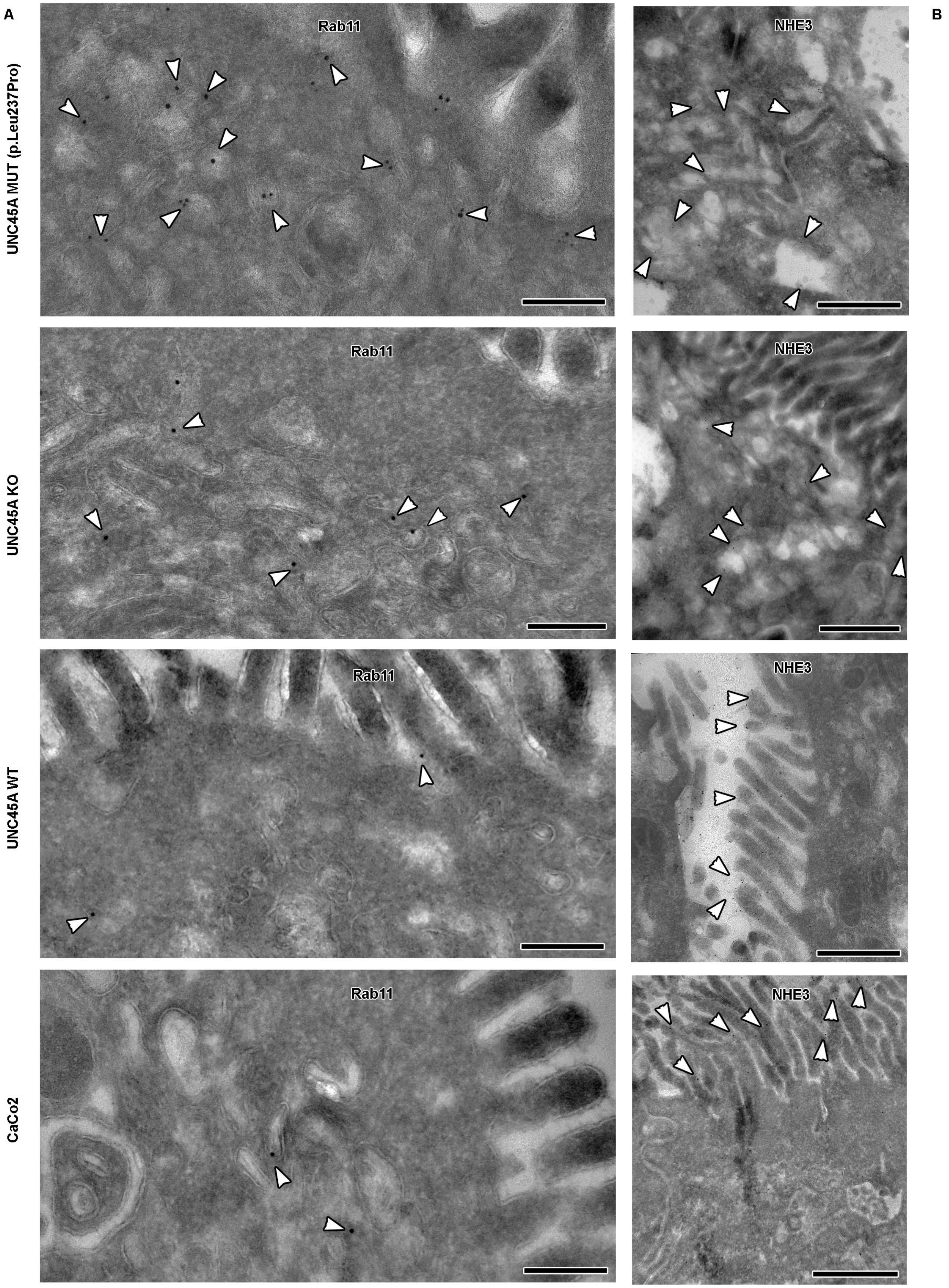

Supplement: Supplemental data [file jciinsight-10-185508-s166.pdf]
