# Supplementary material for: Altered chaperone–nonmuscle myosin II interactions drive pathogenicity of the UNC45A c.710T>C variant in osteo-oto-hepato-enteric syndrome
Source: JCI Insight. 2025 Mar 24;10(6):e185508. doi: 10.1172/jci.insight.185508 (PMC11949031; doi:10.1172/jci.insight.185508)

Full unedited gels for Figure 2A

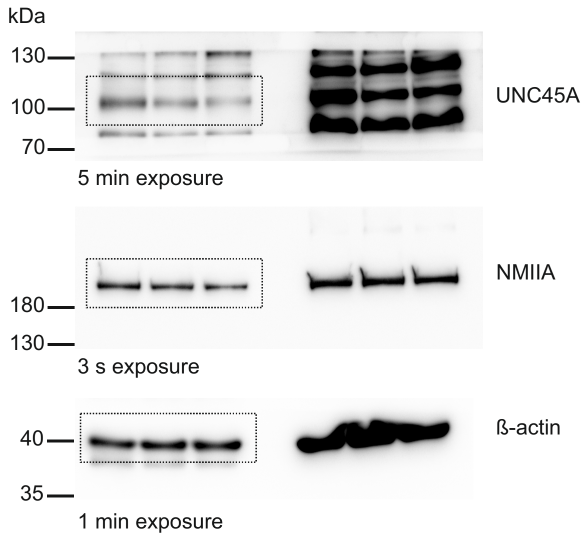

Full unedited gels for Figure 2B

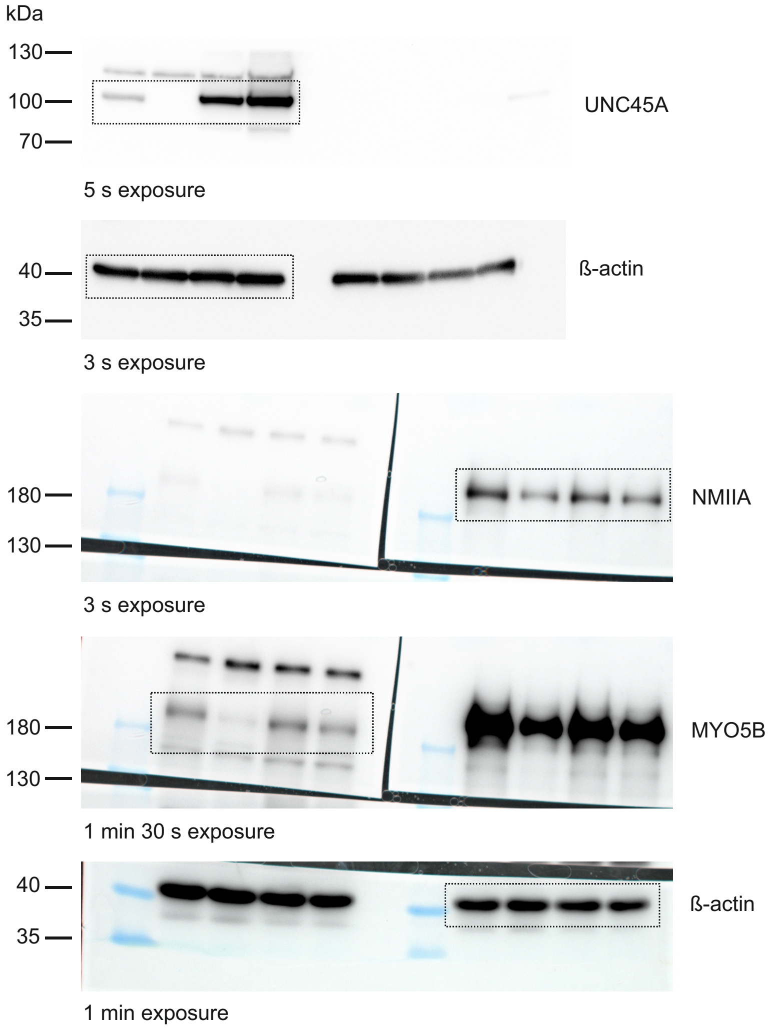

Full unedited gels for Figure 2C

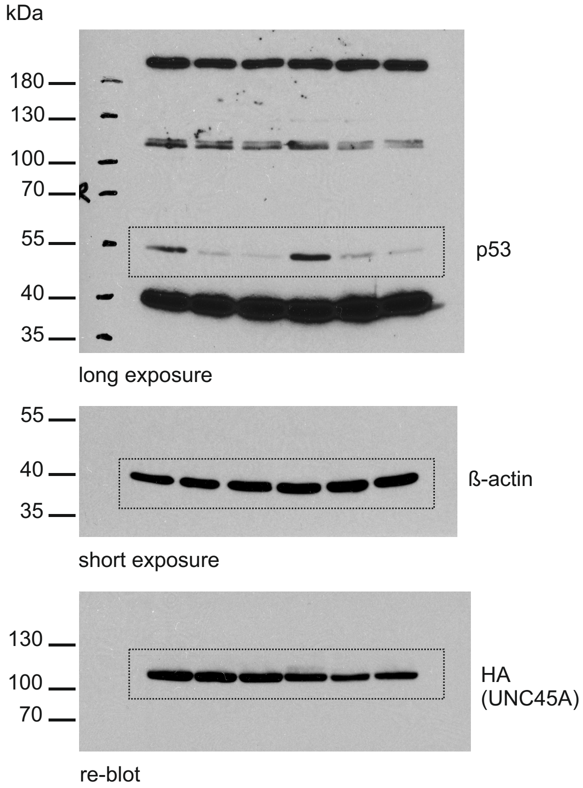

Full unedited gels for Figure 5A

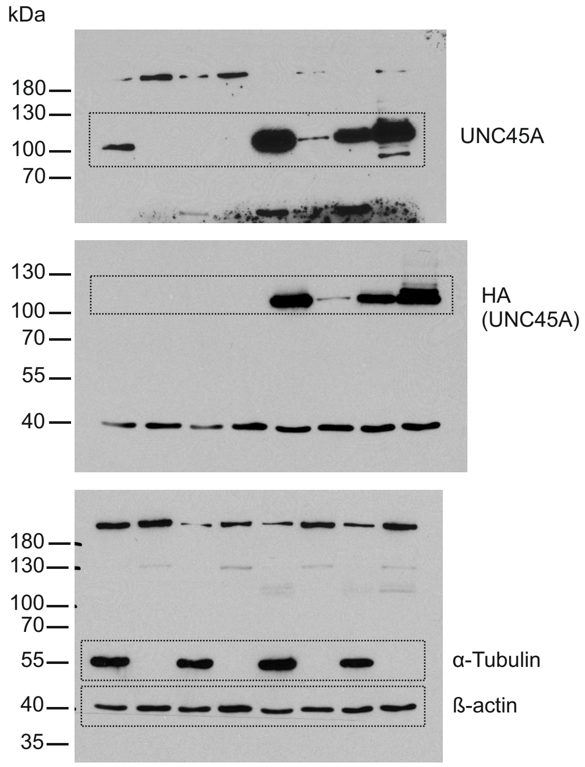

Full unedited gels for Figure 5B (right panel)

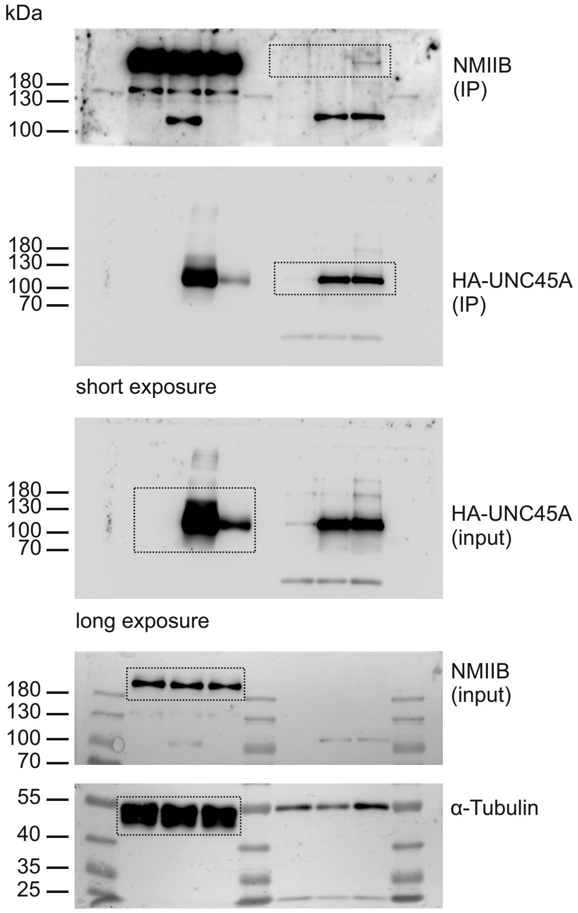

Full unedited gels for Figure 5B (left panel)

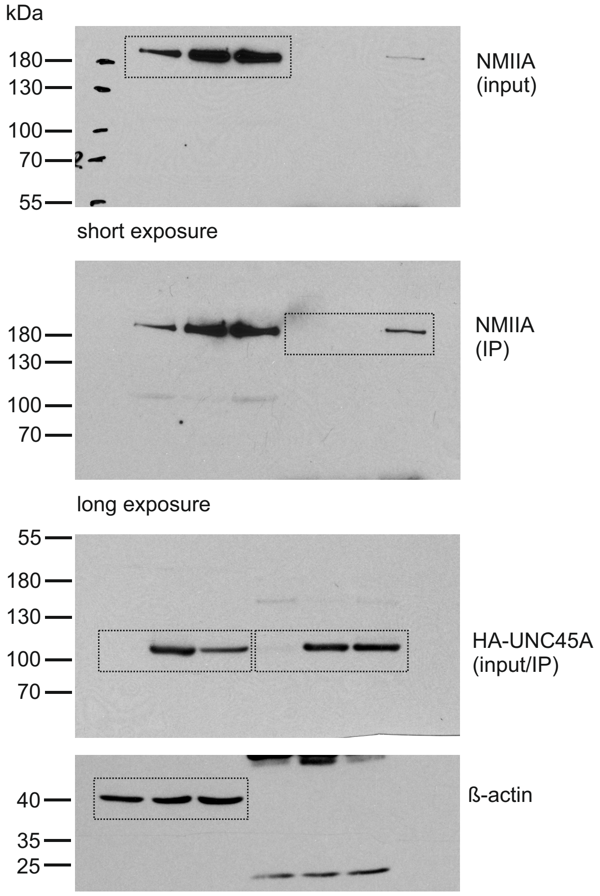

Full unedited gels for Figure 5C (immunoblot panels)

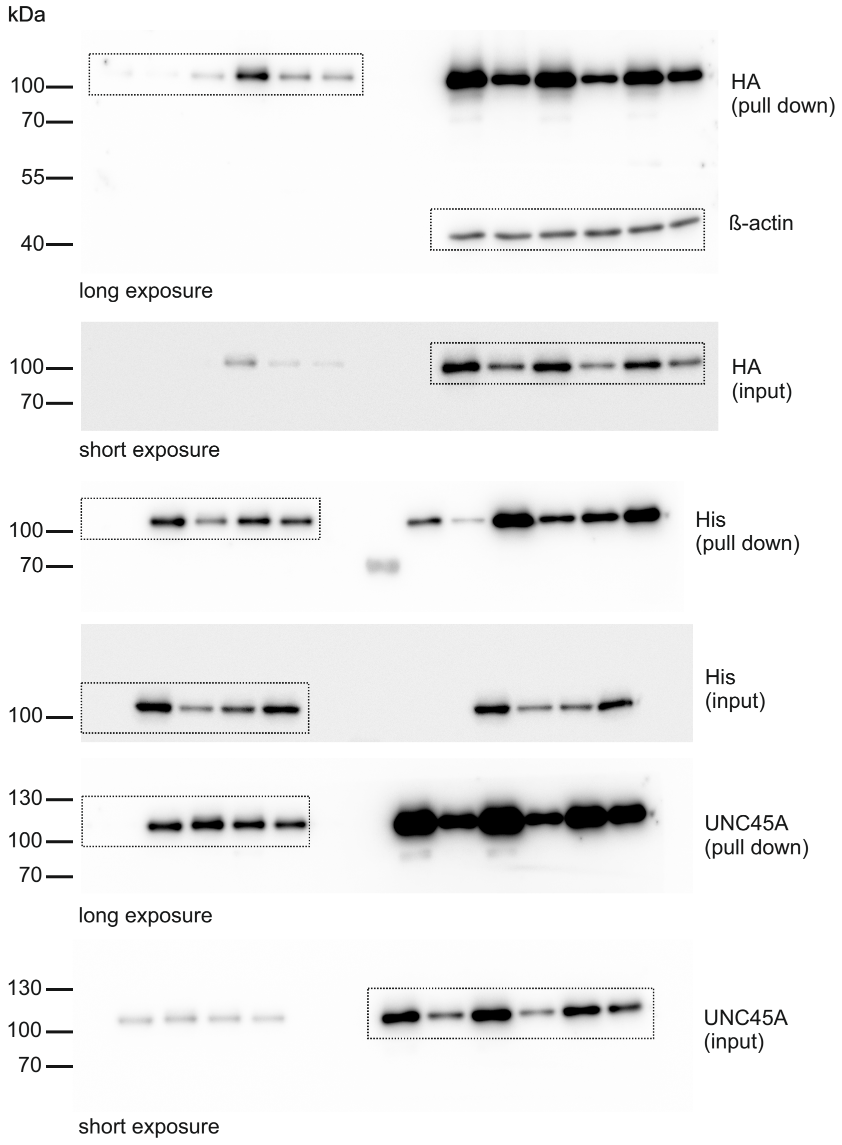

Supplement: Unedited blot and gel images [file jciinsight-10-185508-s167.pdf]
